# Large-Scale Neuromorphic Spiking Array Processors: A Quest to Mimic the Brain

**DOI:** 10.3389/fnins.2018.00891

**Published:** 2018-12-03

**Authors:** Chetan Singh Thakur, Jamal Lottier Molin, Gert Cauwenberghs, Giacomo Indiveri, Kundan Kumar, Ning Qiao, Johannes Schemmel, Runchun Wang, Elisabetta Chicca, Jennifer Olson Hasler, Jae-sun Seo, Shimeng Yu, Yu Cao, André van Schaik, Ralph Etienne-Cummings

**Affiliations:** ^1^Department of Electronic Systems Engineering, Indian Institute of Science, Bangalore, India; ^2^Department of Electrical and Computer Engineering, Johns Hopkins University, Baltimore, MD, United States; ^3^Department of Bioengineering and Institute for Neural Computation, University of California, San Diego, La Jolla, CA, United States; ^4^Institute of Neuroinformatics, University of Zurich and ETH Zurich, Zurich, Switzerland; ^5^Kirchhoff Institute for Physics, University of Heidelberg, Heidelberg, Germany; ^6^The MARCS Institute, Western Sydney University, Kingswood, NSW, Australia; ^7^Cognitive Interaction Technology – Center of Excellence, Bielefeld University, Bielefeld, Germany; ^8^School of Electrical and Computer Engineering, Georgia Institute of Technology, Atlanta, GA, United States; ^9^School of Electrical, Computer and Engineering, Arizona State University, Tempe, AZ, United States

**Keywords:** neuromorphic engineering, large-scale systems, brain-inspired computing, analog sub-threshold, spiking neural emulator

## Abstract

Neuromorphic engineering (NE) encompasses a diverse range of approaches to information processing that are inspired by neurobiological systems, and this feature distinguishes neuromorphic systems from conventional computing systems. The brain has evolved over billions of years to solve difficult engineering problems by using efficient, parallel, low-power computation. The goal of NE is to design systems capable of brain-like computation. Numerous large-scale neuromorphic projects have emerged recently. This interdisciplinary field was listed among the top 10 technology breakthroughs of 2014 by the MIT Technology Review and among the top 10 emerging technologies of 2015 by the World Economic Forum. NE has two-way goals: one, a scientific goal to understand the computational properties of biological neural systems by using models implemented in integrated circuits (ICs); second, an engineering goal to exploit the known properties of biological systems to design and implement efficient devices for engineering applications. Building hardware neural emulators can be extremely useful for simulating large-scale neural models to explain how intelligent behavior arises in the brain. The principal advantages of neuromorphic emulators are that they are highly energy efficient, parallel and distributed, and require a small silicon area. Thus, compared to conventional CPUs, these neuromorphic emulators are beneficial in many engineering applications such as for the porting of deep learning algorithms for various recognitions tasks. In this review article, we describe some of the most significant neuromorphic spiking emulators, compare the different architectures and approaches used by them, illustrate their advantages and drawbacks, and highlight the capabilities that each can deliver to neural modelers. This article focuses on the discussion of large-scale emulators and is a continuation of a previous review of various neural and synapse circuits (Indiveri et al., 2011). We also explore applications where these emulators have been used and discuss some of their promising future applications.

## Introduction

“Building a vast digital simulation of the brain could transform neuroscience and medicine and reveal new ways of making more powerful computers” (Markram et al., 2011). The human brain is by far the most computationally complex, efficient, and robust computing system operating under low-power and small-size constraints. It utilizes over 100 billion neurons and 100 trillion synapses for achieving these specifications. Even the existing supercomputing platforms are unable to demonstrate full cortex simulation in real-time with the complex detailed neuron models. For example, for mouse-scale (2.5 × 10^6^ neurons) cortical simulations, a personal computer uses 40,000 times more power but runs 9,000 times slower than a mouse brain (Eliasmith et al., 2012). The simulation of a human-scale cortical model (2 × 10^10^ neurons), which is the goal of the Human Brain Project, is projected to require an exascale supercomputer (10^18^ flops) and as much power as a quarter-million households (0.5 GW).

The electronics industry is seeking solutions that will enable computers to handle the enormous increase in data processing requirements. Neuromorphic computing is an alternative solution that is inspired by the computational capabilities of the brain. The observation that the brain operates on analog principles of the physics of neural computation that are fundamentally different from digital principles in traditional computing has initiated investigations in the field of neuromorphic engineering (NE) (Mead, 1989a). Silicon neurons are hybrid analog/digital very-large-scale integrated (VLSI) circuits that emulate the electrophysiological behavior of real neurons and synapses. Neural networks using silicon neurons can be emulated directly in hardware rather than being limited to simulations on a general-purpose computer. Such hardware emulations are much more energy efficient than computer simulations, and thus suitable for real-time, large-scale neural emulations. The hardware emulations operate in real-time, and the speed of the network can be independent of the number of neurons or their coupling.

There has been growing interest in neuromorphic processors to perform real-time pattern recognition tasks, such as object recognition and classification, owing to the low energy and silicon area requirements of these systems (Thakur et al., 2017; Wang et al., 2017). These large systems will find application in the next generation of technologies including autonomous cars, drones, and brain-machine interfaces. The neuromorphic chip market is expected to grow exponentially owing to an increasing demand for artificial intelligence and machine learning systems and the need for better-performing ICs and new ways of computation as Moore's law is pushed to its limit (MarketsandMarkets, 2017).

The biological brains of cognitively sophisticated species have evolved to organize their neural sensory information processing with computing machinery that are highly parallel and redundant, yielding great precision and efficiency in pattern recognition and association, despite operating with intrinsically sluggish, noisy, and unreliable individual neural and synaptic components. Brain-inspired neuromorphic processors show great potential for building compact natural signal processing systems, pattern recognition engines, and real-time autonomous agents (Chicca et al., 2014; Merolla et al., 2014; Qiao et al., 2015). Profiting from their massively parallel computing substrate (Qiao et al., 2015) and co-localized memory and computation features, these hardware devices have the potential to solve the von Neumann memory bottleneck problem (Indiveri and Liu, 2015) and to reduce power consumption by several orders of magnitude. Compared to pure digital solutions, mixed-signal neuromorphic processors offer additional advantages in terms of lower silicon area usage, lower power consumption, reduced bandwidth requirements, and additional computational complexity.

Several neuromorphic systems are already being used commercially. For example, Synaptics Inc. develops touchpad and biometric technologies for portable devices, Foveon Inc. develops Complementary Metal Oxide-Semiconductor (CMOS) color imagers (Reiss, 2004), and Chronocam Inc. builds asynchronous time-based image sensors based on the work in Posch et al. (2011). Another product, an artificial retina, is being used in the Logitech Marble trackball, which optically measures the rotation of a ball to move the cursor on a computer screen (Arreguit et al., 1996). The dynamic vision sensor (DVS) by iniLabs Ltd. is another successful neuromorphic product (Lichtsteiner et al., 2008). Table 1 provides a detailed timeline, with major breakthroughs in the field of large-scale brain simulations and neuromorphic hardware.

**Table 1:** Timeline of neuromorphic simulation and hardware.

**References**	**Contributions**
Mead, 1989a	Initiated the field of neuromorphic engineering
Mead, 1989b	Adaptive Retina: among the first biologically inspired silicon retina chip
Mahowald and Douglas, 1991	Silicon Neuron: neuron using subthreshold aVLSI circuitry
Prange and Klar, 1993	BIONIC: an emulator with simulation of 16 neurons with 16 synapses
Yasunaga et al., 1990	LSI composed on 576 digital neurons
Wolpert and Micheli-Tzanakou, 1996	Modeling nerve networks based on the I&F model *in silicon*
Jahnke et al., 1996	NESPINN: SIMD/dataflow architecture for a neuro-computer
Schoenauer et al., 1999	MASPINN: a neuro-accelerator for spiking neural networks
Wolff et al., 1999	ParSPIKE: a DSP accelerator simulating large spiking neural networks
Schoenauer et al., 2002	NeuroPipe-Chip: a digital neuro-processor for spiking neural networks
Furber et al., 2006	High-performance computing for systems of spiking neurons
Markram, 2006	Blue Brain Project: large-scale simulation of the brain at cellular level
Boahen, 2006	Neurogrid: emulating a million neurons in the cortex
Koickal et al., 2007	aVLSI adaptive neuromorphic olfaction chip
Djurfeldt et al., 2008	Brain-scale computer simulation of the neocortex on the IBM Blue Gene
Maguire et al., 2007	BenNuey: platform comprises up to 18 M neurons and 18 M synapses
Izhikevich and Edelman, 2008	Simulation of thalamocortical with 10^11^ neuron and 10^15^ synapses
Ananthanarayanan et al., 2009	Cortical simulations with 10^9^ neurons, 10^13^ synapses
Serrano-Gotarredona et al., 2009	CAVIAR: a 45 k neuron 5 M synapse 12 G connects/s AER hardware
Schemmel et al., 2008, 2010	BrainScales: a wafer-scale neuromorphic hardware
Seo et al., 2011	CMOS neuromorphic chip with 256 neurons and 64 K synapses
Cassidy et al., 2011	EU SCANDLE: one-million-neuron, single FPGA neuromorphic system
Moore et al., 2012	Bluehive project: simulation with 256 k neurons and 256 M synapses
Zamarreno-Ramos et al., 2013	AER system with 64 processors, 262 k neurons, and 32 M synapses
Furber et al., 2014	SpiNNaker: Digital neuromorphic chip with multicore System-on-Chip
Merolla et al., 2014	TrueNorth: IBM introduces the TrueNorth “neurosynaptic chip”
Benjamin et al., 2014	Neurogrid: A mixed-analog-digital large-scale neuromorphic simulator.
Park et al., 2014	IFAT: neuromorphic processor with 65 k-neuron I&F array transceiver
Wang et al., 2014	An FPGA framework simulating 1.5 million LIF neurons in real time
Qiao et al., 2015	A spiking neuromorphic processor with 256 neurons and 128 K synapses
Park et al., 2017	HiFAT-IFAT: reconfigurable large-scale neuromorphic systems
Cheung et al., 2016	Neuromorphic processor capable of simulating 400 k neurons in real-time
Pani et al., 2017	An FPGA platform with up to 1,440 Izhikevich neurons
Moradi et al., 2018	DYNAP-SEL: neuromorphic mixed-signal processor with self-learning
Davies et al., 2018	Loihi: Intel neuromorphic chip with “self-learning chip”
Wang and van Schaik, 2018; Wang et al., 2018	DeepSouth: cortex simulator up to 2.6 billion LIF neurons

In this work, we describe a wide range of neural processors based on different neural design strategies and synapses that range from current-mode, sub-threshold to voltage-mode, switched-capacitor designs. Moreover, we will discuss the advantages and strengths of each system and their potential applications.

## Integrate-And-Fire Array Transceiver (iFAT)

The Integrate-and-Fire Array Transceiver (IFAT) is a mixed-mode VLSI-based neural array with reconfigurable, weighted synapses/connectivity. In its original design, it is comprised of the array of mixed-mode VLSI neurons, an LUT (look-up table), and AER (Address Event Representation) architecture. The AER architecture is used for the receiver and transmitter. The AER communication protocol is an event-based, asynchronous protocol. Addresses are inputs to the chip (address-events, AEs). The addresses represent the neuron receiving the input event/spike. When a neuron outputs an event, it outputs the address (output AE) of the neuron emitting the event/spike. The LUT holds the information on how the network is connected. It consists of the corresponding destination address(es) (post-synaptic events) for each incoming AE (pre-synaptic event). For each connection, there is a corresponding weight signifying the strength of the post-synaptic event. The larger the weight, the more charge integrated onto the membrane capacitance of the destination neuron receiving the post-synaptic event. There is also a polarity bit corresponding to each synapse signifying an inhibitory or excitatory post-synaptic event. The original design of the IFAT utilized probabilistic synapses (Goldberg et al., 2001). The weights were represented by a probability. As events were received, the probability of the neuron receiving the event was represented as the weight. The neuron circuit used was essentially a membrane capacitor coupled to a comparator and a synapse implemented as a transmission gate and charge pump. The next generation of the IFAT used conductance-based synapses (Vogelstein et al., 2007a). Instead of representing weights as probabilities, a switch-cap circuit was used. The weight then represented the synapse capacitance, and therefore, was proportional to the amount of charge integrated onto the membrane capacitance.

In sections MNIFAT: (Mihalas–Niebur and Integrate-and-Fire Array Transceiver) and HiAER-IFAT: Hierarchical Address-Event Routing (HiAER) Integrate-and-Fire Array Transceivers (IFAT), two novel variants of the IFAT will be depicted: MNIFAT and HiAER IFAT.

### MNIFAT: (Mihalas–Niebur and Integrate-And-Fire Array Transceiver)

This section describes novel integrate-and-fire array transceiver (IFAT) neural array (MNIFAT), which consists of 2,040 Mihalas–Niebur (M–N) neurons developed in the lab of Ralph Etienne-Cummings at the Johns Hopkins University. The M–N neuron circuit design used in this array was shown to produce nine prominent spiking behaviors using an adaptive threshold. Each of these M–N neurons were designed to have the capability to operate as two independent integrate-and-fire (I&F) neurons. This resulted in 2,040 M–N neurons and 4,080 leaky I&F neurons. This neural array was implemented in 0.5 μm CMOS technology with a 5 V nominal power supply voltage (Lichtsteiner et al., 2008). Each I&F consumes an area of 1,495 μm^2^, while the neural array dissipates an average of 360 pJ of energy per synaptic event at 5 V. This novel neural array design consumes considerably less power and area per neuron than other neural arrays designed in CMOS technology of comparable feature size. Furthermore, the nature of the design allows for more controlled mismatch between neurons.

#### Neural Array Design

**Figure 1:**
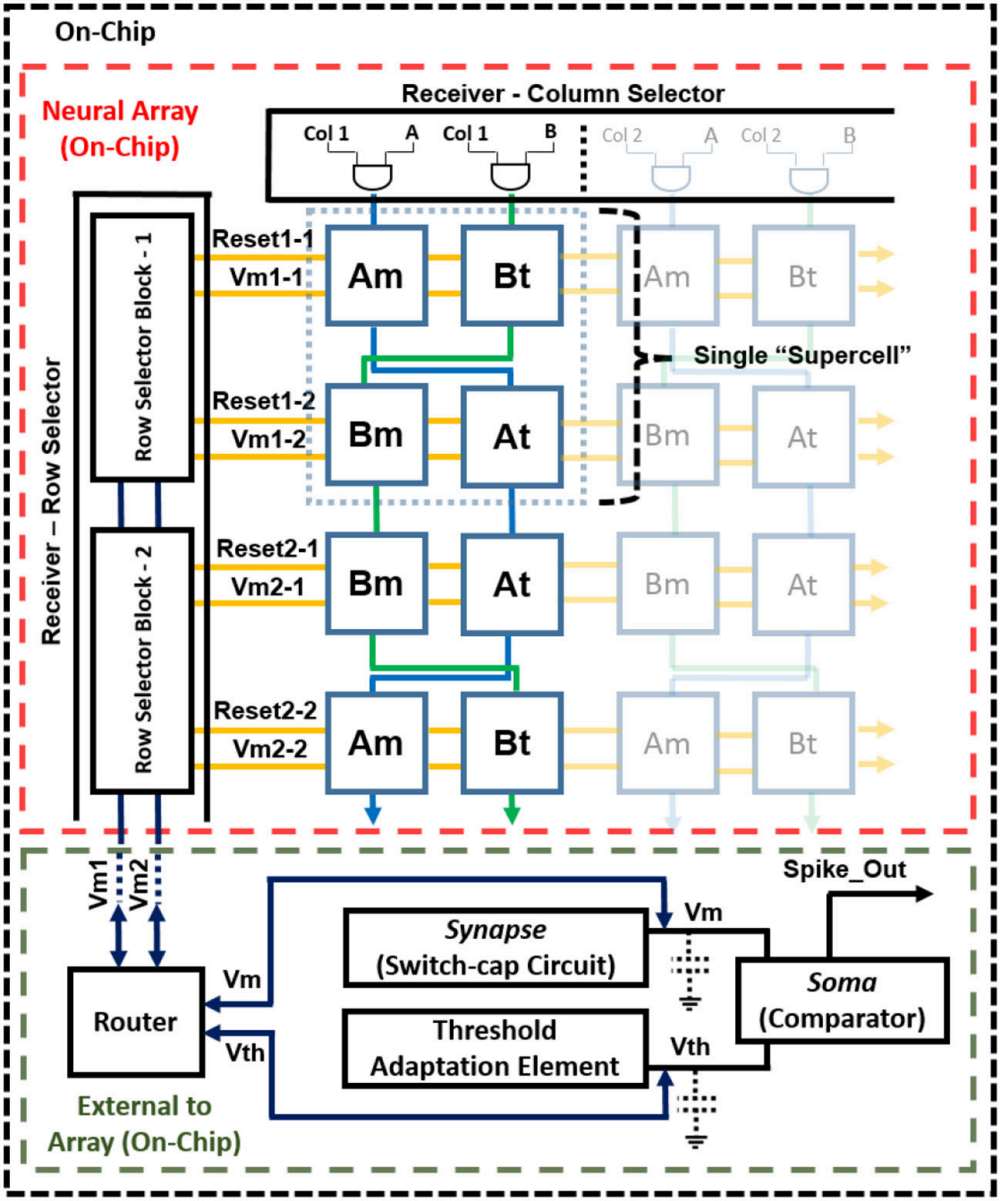
Mihalas–Niebur neural array design.

The complete block diagram of the neuron array chip is shown in Figure 1. It was implemented with the aim to maximize the neuron array density, minimize power consumption, and reduce mismatch due to process variation. This is achieved by utilizing a single membrane synapse (switch-capacitor circuit) and soma (comparator) shared by all neurons in the array. The connection between the neurons is reconfigurable via an off-chip LUT. Pre-synaptic events are sent first through the LUT where the destination addresses and synaptic strengths are stored. Post-synaptic events are then sent to the chip. These events are sent as AEs along a shared address bus decoded by the row decoder and column decoder on-chip. The incoming address corresponds to a single neuron in the array.

The neuron array is made up of supercells, each containing four cells, labeled *A*_*m*_, *A*_*t*_, *B*_*m*_, and *B*_*t*_. Each supercell contains two M–N neurons, one using *A*_*m*_ and *A*_*t*_ cells, and the second using Bm and Bt cells. Each of these M–N neurons can also operate as two independent, leaky (I&F) neurons, resulting in a total of four leaky I&F neurons (*A*_*m*_, *A*_*t*_, *B*_*m*_, and *B*_*t*_). Incoming AE selects the supercell in the array and consists of two additional bits for selecting one of the two M–N neurons (A or B) within the supercell, or one of the four cells when operating as I&F neurons. Finally, the voltage across the storage capacitance for both the membrane cell and threshold cell is buffered to the processor via the router (*V*_*m*_1 − X and *V*_*m*_2 − X, where X is the row selected). The router is used for selecting which voltage (from the membrane cell or threshold cell) is buffered to the processor as the membrane voltage and/or threshold voltage, depending on the mode selected (M–N mode or I&F mode). This router is necessary for allowing the voltage from the threshold (*A*_*t*_ or *B*_*t*_) cell to be used as the membrane voltage when in I&F mode. After the selected neuron cell(s) buffer their stored voltage to the external capacitances *C*_*m*_ and *C*_*t*_, the synaptic event is applied and the new voltage is buffered back to the same selected cells that received the event. The synapse and threshold adaptation elements execute the neuron dynamics as events are received. If the membrane voltage exceeds the threshold voltage, there is a single comparator (soma) that outputs a logic high (event).

An output arbiter/transmitter is not necessary in the design considering that a neuron only fires when it receives an event. The single output signal always corresponds to the neuron that receives the incoming event. Having a single comparator not only reduces power consumption but also reduces the required number of pads for digital output. In this design, the speed is compromised (for low-power and low-area) due to the time necessary to read and write to and from the neuron. However, a maximum input event rate of ~1 MHz can still be achieved for proper operation.

#### Mihalas–Niebur (M–N) Neuron Model and Circuit Implementation

Each cell pair (*A*_*m*_*/A*_*t*_ and *B*_*m*_*/B*_*t*_) in this neural array models the M–N neuron dynamics (Mihalaş and Niebur, 2009). In its original form, it uses linear differential equations and parameters with biological facsimiles. It consists of an adaptive threshold and was shown to be capable of modeling all biologically relevant neuron behaviors. The differential equations for CMOS implementation of M–N model are as follows:

(1) $$\begin{array}{r} V_{m}\prime (t)=\frac{g_{l}^{m}}{C_{m}}(V_{r}-V_{m}(t)) \end{array}$$

(2) $$\begin{array}{r} \theta^{\prime }\left(t\right)=\frac{g_{l}^{t}}{C_{t}}\left(\theta_{r}-\theta \left(t\right)\right) \end{array}$$

(3) $$\begin{array}{r} V_{m}\left(t+1\right)=V_{m}\left(t\right)+\frac{C_{s}^{m}}{C_{m}}\left(E_{m}-V_{m}\left(t\right)\right) \end{array}$$

(4) $$\begin{array}{r} \theta \left(t+1\right)=\theta \left(t\right)+\frac{C_{s}^{t}}{C_{t}}\left(V_{m}\left(t\right)-V_{r}\right) \end{array}$$

and,

(5) $$\begin{array}{r} g_{l}^{m}=\frac{1}{r_{l}^{m}}=f_{l}^{m}C_{l} \end{array}$$

(6) $$\begin{array}{r} g_{l}^{t}=\frac{1}{r_{l}^{t}}=f_{l}^{t}C_{l} \end{array}$$

Equations (3) and (4) model the change in membrane potential (*V*_*m*_) and threshold potential (θ) at each time step as the neuron receives an input. $C_{s}^{m}$ and $C_{s}^{t}$ are the switch-capacitor capacitance depicting the synapse conductance or threshold adaptation conductance, respectively. *C*_*m*_ and *C*_*t*_ are the storage capacitance for the membrane and threshold cells, respectively. *E*_*m*_ is the synaptic driving potential. Equations (1) and (2) model the leakage dynamics, independent of synaptic connections. $g_{l}^{m}$ and $g_{l}^{t}$ are the leakage conductances for the membrane and threshold and are dependent on the clock frequency, $f_{l}^{m}$ and $f_{l}^{t}$. The update rules for this M–N neuron model are as follows:

(7) $$\begin{array}{r} V_{m}\left(t\right)\leftarrow V_{r} \end{array}$$

(8) $$\begin{array}{r} \theta \left(t\right)\leftarrow \left\{if\;\theta \left(t\right)>V_{m}\left(t\right),\;\theta \left(t\right)\;;\;if\;\theta \left(t\right)\leq V_{m}\left(t\right),\;\theta_{r}\right\} \end{array}$$

#### Neuron Cell Circuit

**Figure 2:**
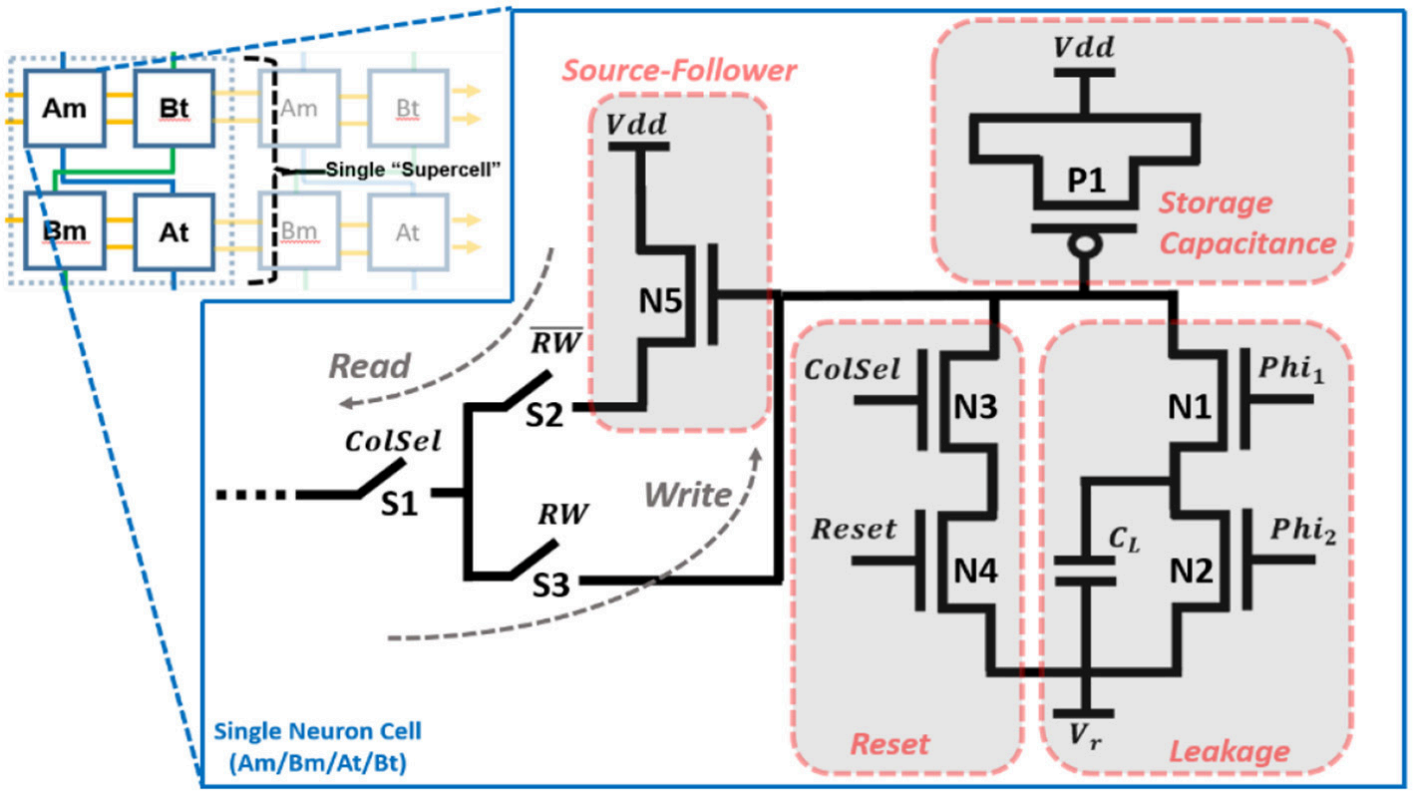
Single neuron cell design.

The neuron cell circuit is shown in Figure 2. The PMOS transistor, P1, is the storage capacitance (~440 fF), *C*_*m*_ *or C*_*t*_ (depending on whether the cell is being used to model the membrane or threshold dynamics) implemented as a MOS capacitor with its source and drain tied to Vdd. Transistors N1 and N2 model the leakage (Equations 1 and 2) via a switch capacitor circuit with Phi1 and Phi2 pulses at a rate of $f_{l}^{m,t}$ (also, *C*_*l*_≪*C*_*m*_). Transistors N3 and N4 allow for resetting the neuron when selected (ColSel = “1”). Transistor N5 forms a source-follower when coupled with a globally shared variable resistance located in the processor of the neural array. It is implemented as an NMOS transistor with a voltage bias (*V*_*b*_). In read mode (RW = “0”), switch S2 is closed such that the voltage across the storage capacitance is buffered to an equivalent capacitance coupled to the synapse and/or threshold adaptation element. In write mode (RW = “1”), switch S3 is closed such that the new voltage from the synapse/threshold elements (after an event is received) is buffered to the storage capacitance.

#### Synapse and Threshold Adaptation Circuits

**Figure 3:**
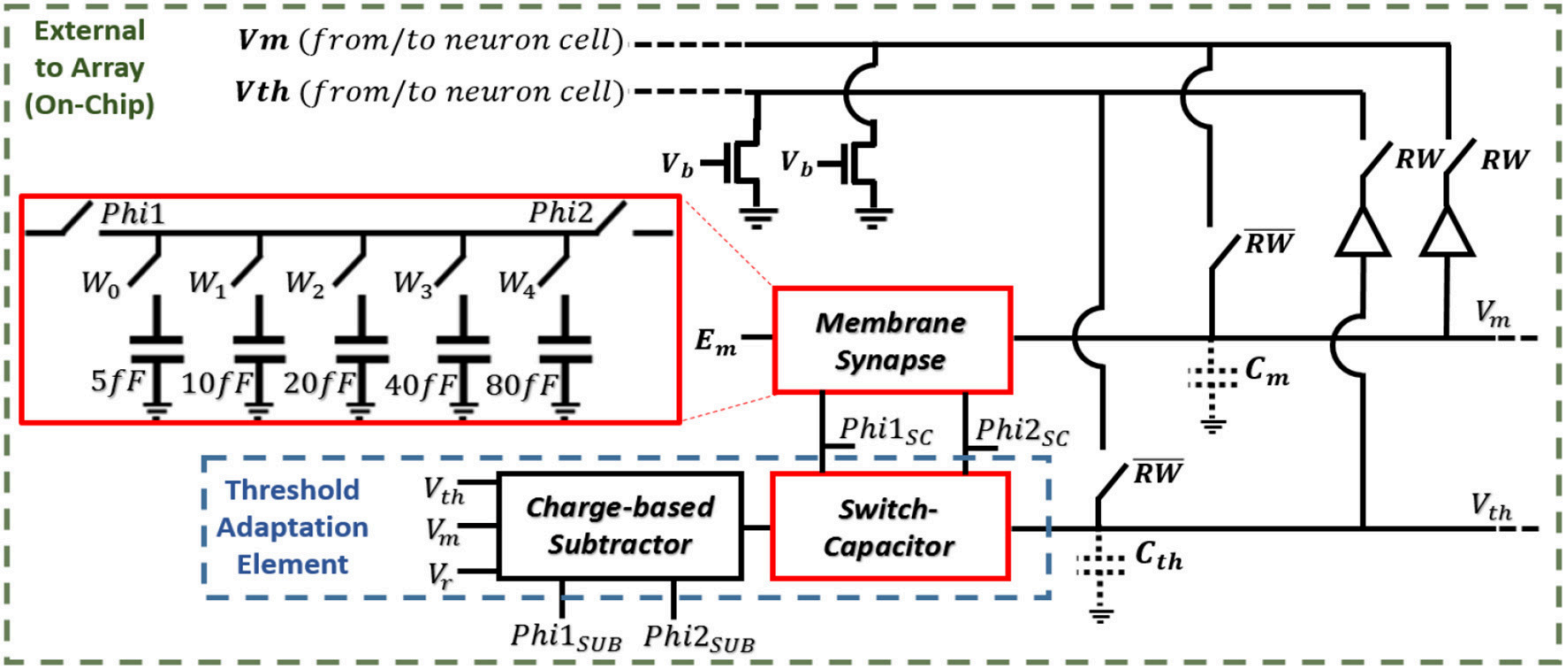
Block diagram of the processor including the synapse and threshold adaptation circuits.

The schematic for modeling the neuron dynamics can be seen in Figure 3. When a neuron receives an event, RW = “0,” and the neuron's cell is selected, and its stored membrane voltage is buffered to the capacitance *C*_*m*_. In the same manner, if in M–N mode, the threshold voltage is buffered to *C*_*t*_. The Phi1_SC_ and Phi2_SC_ pulses are then applied (off-chip), adding (excitatory event), or removing (inhibitory event) charge to *C*_*m*_ via the synapse using a switch-capacitor circuit. A second, identical switch-capacitor circuit is used for implementing the threshold adaptation dynamics. As a neuron receives events, the same Phi1_SC_ and Phi2_SC_ pulses are applied to the threshold adaptation switch-capacitor circuit that adds or removes charge to *C*_*t*_. The new voltage is then buffered (RW = “1”) back to the neuron cells for storing the new membrane voltage (as well as the threshold voltage if in M–N mode). When using each neuron independently as leaky I&F neurons, the threshold adaptive element is bypassed and an externally applied fixed threshold voltage is used. A charge-based subtractoris used in the threshold adaptation circuit for computing *V*_*th*_+(*V*_*m*_−*V*_*r*_) in modeling Equation (4). This subtraction output is the driving potential for the threshold switch-capacitor circuit. An externally applied voltage, *E*_*m*_, is the synaptic driving potential for the membrane synapse and is used for modeling Equation (3). Finally, the comparator outputs an event when the membrane voltage exceeds the threshold voltage. An external reset signal for both the neuron cell modeling the membrane voltage and cell modeling the threshold voltage is activated for the selected neuron (via Reset1-X and Reset2-X) when a spike is outputted.

#### Results

A single neuron cell in this array has dimensions of 41.7 × 35.84 μm. It consumes only 62.3% of the area consumed by a single neuron cell in Vogelstein et al. (2007a), also designed in a 0.5 μm process. This work achieves 668.9 I&F neurons/mm^2^, Vogelstein et al. (2007a) achieves only 416.7 neurons/mm^2^, and Moradi and Indiveri (2011) achieves only 387.1 neurons/mm^2^. The number of neurons/mm^2^ can be further increased by optimizing the layout of the neuron cell and implementing in smaller feature-size technology.

The mismatch (due to process variations) across the neuron array was analyzed, as well as the output event to input event ratio for a fixed synaptic weight and an input event rate of 1 MHz was observed for each neuron in the array. With this fixed synaptic weight, the 2,040 M–N neurons have a mean output to input event ratio of 0.0208 ±1.22e-5. In the second mode of operation, the 4,080 I&F neurons have a mean output to input event ratio of 0.0222 ±5.57e-5. The results were also compared with a similar experiment performed in the 0.5-μm conductance-based IFAT in Vogelstein et al. (2007a) (Table 2). The design shows significantly less deviation. Small amounts of mismatch can be taken advantage of in applications that require stochasticity. However, for those spike-based applications that do not benefit from mismatch, in this neural array, it is more controlled. This again is a result of utilizing a single, shared synapse, comparator, and threshold adaptive element for all neurons in the array. The mismatch between neurons is only due to the devices within the neuron cell itself.

**Table 2:** Array characterization showing output events per input event.

**Neural array**	**Mean ratio (μ)**	**Standard deviation (σ)**	**No. of neurons**
IFAT	0.0222	±5.57e-5	4,080
Vogelstein et al., 2007a	0.0210	±1.70e-3	2,400

Another design goal was to minimize power consumption. At an input event rate of 1 MHz, the average power consumption was 360 μW at 5.0 V power supply. A better representation of the power consumption is energy per incoming event. From these measurements, this chip consumes 360 pJ of energy per synaptic event. A comparison with other state-of-the-art neural array chips can be seen in Table 3. Compared to those chips designed in 500 nm (Vogelstein et al., 2007a) and 800 nm (Indiveri et al., 2006) technology, a significant reduction in energy per synaptic event was seen. Due to complications in the circuit board, the low-voltage operation could not be measured. However, the proper operation at 1.0 V (at slower speeds) was validated using simulations. Assuming dynamic energy scales with V^2^ (capacitance remains the same), the energy per synaptic event was estimated as ~14.4 pJ at 1.0 V. These results are promising, as these specifications will be even further optimized by designing in smaller feature-size technology. Table 3 summarizes the specifications achieved from this chip.

**Table 3:** Measured (^*^estimated) chip results.

**Process (*nm*)**	**Vdd supply (*V*)**	**(I&F) neuron density (*neurons*/*mm*^2^)**	**Neuron area (*μm*^2^)**	**Energy/Event (*pJ*)**
500	5.0	669	1, 495	360
55^*^	1.2	55, 298	18.1	20.7

#### Application

The IFAT system can be employed for mimicking various biological systems, considering the reconfigurability feature of the system. Furthermore, the use of the M–N neuron model allows for simulation of various spiking behaviors, which in turn allows for a wider range of neurological dynamics to be implemented. Aside from neurological simulation, this IFAT system can be utilized for performing visual processing tasks. The reconfigurability of the synaptic connections between neurons and the one-to-many capability allows for linear filtering, including edge and smoothing operators. The reconfigurability is also dynamic such that it can implement image dewarping operations. As events/spikes enter the system, they can be projected to new locations in the neural array based on camera rotation and translation. These image processing tasks make our system ideal for low-power visual preprocessing. Recent technology, including autonomous drones and self-driving cars require complex visual processing. Utilizing this IFAT system to perform preprocessing, feed-forward visual tasks would prove beneficial for such advanced technology performing object recognition and classification tasks.

### HiAER-IFAT: Hierarchical Address-Event Routing (HiAER) Integrate-And-Fire Array Transceivers (IFAT)

Hierarchical address-event routing integrate-and-fire array transceiver (HiAER-IFAT) provides a multiscale tree-based extension of AER synaptic routing for dynamically reconfigurable long-range synaptic connectivity in neuromorphic computing systems, developed in the lab of Gert Cauwenberghs at the University of California San Diego. A major challenge in scaling up neuromorphic computing to the dimensions and complexity of the human brain, a necessary endeavor toward bio-inspired general artificial intelligence approaching human-level natural intelligence, is to accommodate massive flexible long-range synaptic connectivity between neurons across the network in highly efficient and scalable manner. Meeting this challenge calls for a multi-scale system architecture, akin to the organization of gray and white matter distinctly serving local compute and global communication functions in the biological brain, that combines highly efficient, dense, local synaptic connectivity with highly flexible, reconfigurable, sparse, long-range connectivity.

**Figure 4:**
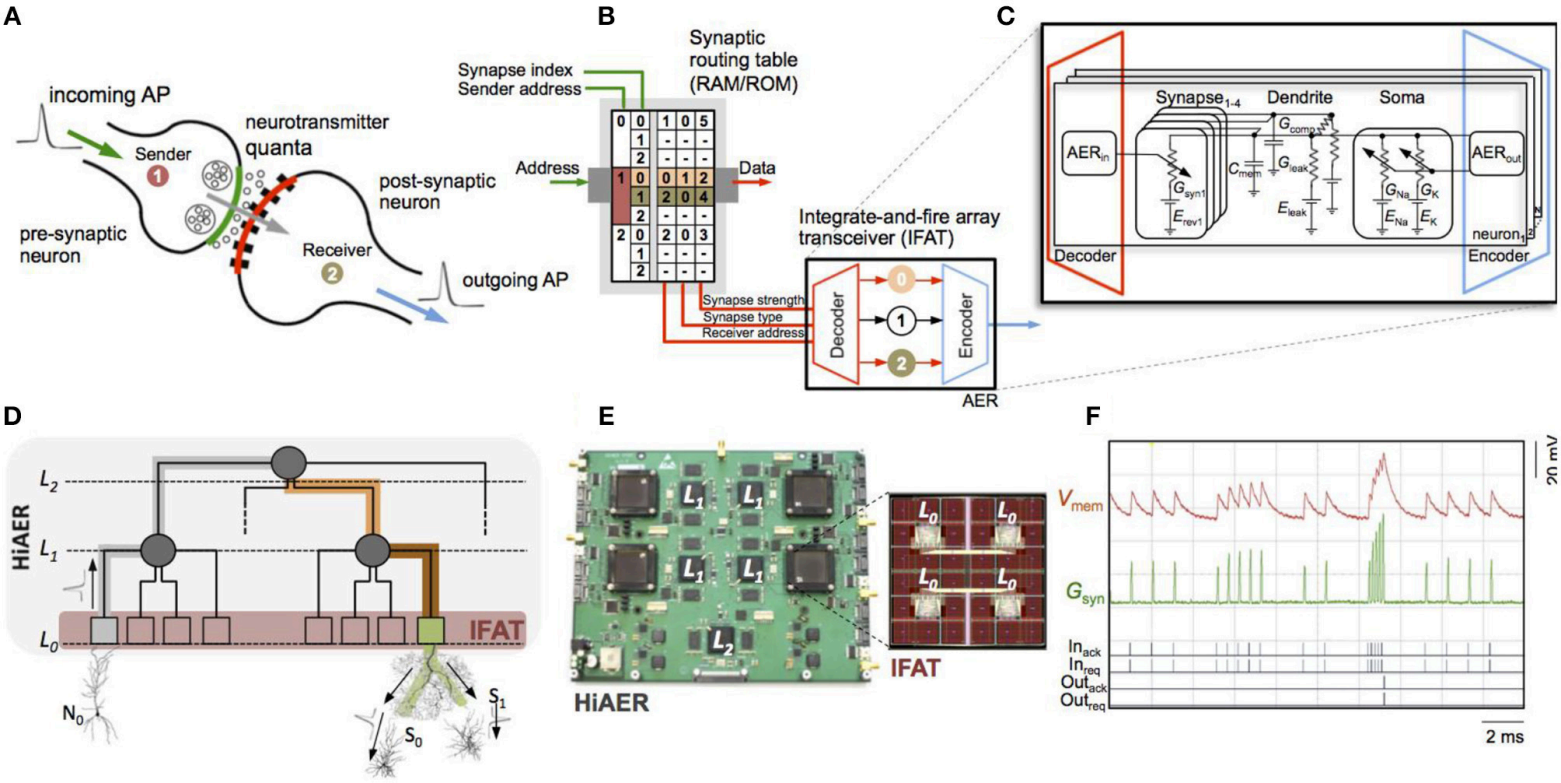
Hierarchical address-event routing (HiAER) integrate-and-fire array transceiver (IFAT) for scalable and reconfigurable neuromorphic neocortical processing (Broccard et al., 2017; Park et al., 2017). **(A)** Biophysical model of neural and synaptic dynamics. **(B)** Dynamically reconfigurable synaptic connectivity is implemented across IFAT arrays of addressable neurons by routing neural spike events locally through DRAM synaptic routing tables (Vogelstein et al., 2007a,b). **(C)** Each neural cell models conductance-based membrane dynamics in proximal and distal compartments for synaptic input with programmable axonal delay, conductance, and reversal potential (Yu et al., 2012; Park et al., 2014). **(D)** Multiscale global connectivity through a hierarchical network of HiAER routing nodes (Joshi et al., 2010). **(E)** HiAER-IFAT board with 4 IFAT custom silicon microchips, serving 256 k neurons and 256 M synapses, and spanning 3 HiAER levels (L0-L2) in connectivity hierarchy (Park et al., 2017). **(F)** The IFAT neural array multiplexes and integrates (top traces) incoming spike synaptic events to produce outgoing spike neural events (bottom traces) (Yu et al., 2012). The most recent IFAT microchip-measured energy consumption is 22 pJ per spike event (Park et al., 2014), several orders of magnitude more efficient than emulation on CPU/GPU platforms.

Efforts toward this objective for large-scale emulation of neocortical vision have resulted in event-driven spiking neural arrays with dynamically reconfigurable synaptic connections in a multi-scale hierarchy of compute and communication nodes abstracting such gray and white matter organization in the visual cortex (Figure 4) (Park et al., 2012, 2014, 2017). Hierarchical address-event routing (HiAER) offers scalable long-range neural event communication tailored to locally dense and globally sparse synaptic connectivity (Joshi et al., 2010; Park et al., 2017), while IFAT CMOS neural arrays with up to 65 k neurons integrated on a single chip (Vogelstein et al., 2007a,b; Yu et al., 2012) offer low-power implementation of continuous-time analog membrane dynamics at energy levels down to 22 pJ/spike (Park et al., 2014).

The energy efficiency of such large-scale neuromorphic computing systems is limited primarily by the energy costs of external memory access as needed for table lookup of fully reconfigurable, sparse synaptic connectivity. Greater densities and energy efficiencies can be obtained by integrating them to replace the core of the external DRAM memory lookup in HiAER-IFAT flexible cognitive learning and inference systems with nano-scale memristor synapse arrays vertically interfacing with neuron arrays (Kuzum et al., 2012), and further optimizing the vertically integrated circuits (ICs) toward sub-pJ/spike overall energy efficiency in neocortical neural and synaptic computation and communication (Hamdioui et al., 2017).

#### Application

Online unsupervised learning with event-driven contrastive divergence (Neftci et al., 2014) using a wake-sleep modulated form of biologically inspired spike-timing dependent plasticity (Bi and Poo, 1998) produces generative models of probabilistic spike-based neural representations, offering a means to perform Bayesian inference in deep networks of large-scale spiking neuromorphic systems. The algorithmic advances in hierarchical deep learning and Bayesian inference harness the inherent stochastic nature of the computational primitives at the device level, such as the superior generalization and efficiency of learning of drop-connect emulating the pervasive stochastic nature of biological neurons and synapses (Al-Shedivat et al., 2015; Naous et al., 2016) by the Spiking Synaptic Sampling Machine (S3M) (Eryilmaz et al., 2016; Neftci et al., 2016). Target applications range from large-scale simulation of cortical models for computational neuroscience, and acceleration of spike-based learning methods for neuromorphic computing adaptive intelligence.

## DeepSouth

DeepSouth, the cortex emulator was designed for simulating large and structurally connected spiking neural networks in the lab of André van Schaik at the MARCS Institute, Western Sydney University, Australia. Inspired by observations from neurobiology, the fundamental computing unit is called a minicolumn, which consists of 100 neurons. Simulating large-scale, fully connected networks needs prohibitively large memory to store LUTs for point-to-point connections. Instead, they came up with a novel architecture, based on the structural connectivity in the neocortex, such that all the required parameters and connections can be stored in on-chip memory. The cortex emulator can be easily reconfigured for simulating different neural networks without any change in hardware structure by programming the memory. A hierarchical communication scheme allows one neuron to have a fan-out of up to 200 k neurons. As a proof-of-concept, an implementation on a Terasic DE5 development kit was able to simulate upto 2.6 billion leaky-integrate-and-fire (LIF) neurons in real time. When running at five times slower than real time, it can simulate upto 12.8 billion LIF neurons, which is the maximum network size on the chosen FPGA board, due to memory limitations. Larger networks could be implemented on larger FPGA boards with more external memory.

### Strategy

#### Modular Structure

**Figure 5:**
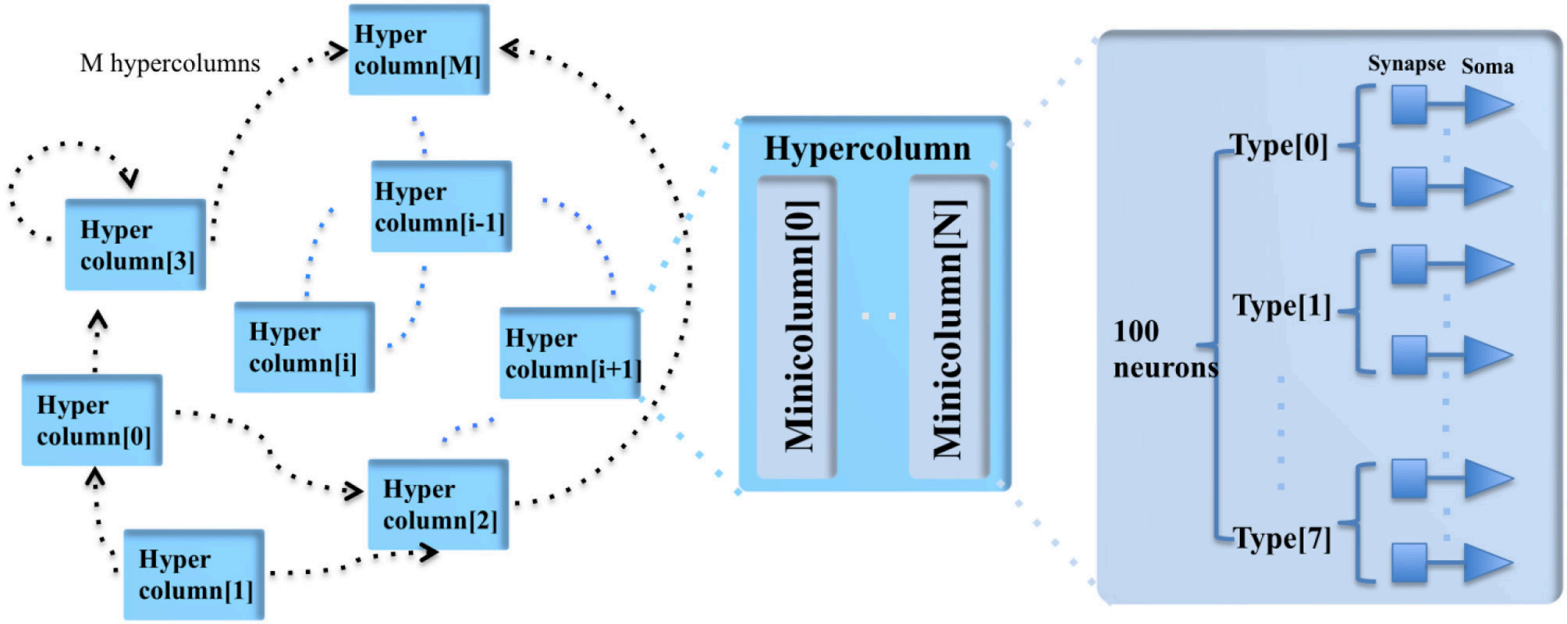
The modular structure of the cortex emulator. The basic building block of the cortex emulator is the minicolumn, which consists of up to eight different types of heterogeneous neurons (100 in total). The functional building block is the hypercolumn, which can have up to 128 minicolumns. The connections are hierarchical: hypercolumn-level connections, minicolumn-level connections, and neuron-level connections.

The cortex is a structure composed of a large number of repeated units, neurons and synapses, each with several sub-types, as shown in Figure 5. A minicolumn is a vertical column of cortex with about 100 neurons and stretches through all layers of the cortex (Buxhoeveden and Casanova, 2002a). Each minicolumn contains excitatory neurons, mainly pyramidal and stellate cells, inhibitory inter neurons, and many internal and external connections. The minicolumn is often considered to be both a functional and anatomical unit of the cortex (Buxhoeveden and Casanova, 2002b), and DeepSouth uses this minicolumn with 100 neurons as the basic building block of the cortex emulator. The minicolumn in the cortex emulator is designed to have up to eight different programmable types of neurons. Note, the neuron types do not necessarily correspond to the cortical layers, but can be configured as such.

In the mammalian cortex, minicolumns are grouped into modules called hypercolumns (Hubel and Wiesel, 1977). These are the building blocks for complex models of various areas of the cortex (Johansson and Lansner, 2007). The hypercolumn acts as a functional grouping for the emulator. The hypercolumn in the cortex emulator is designed to have up to 128 minicolumns. Like the minicolumns, the parameters of the hypercolumns are designed to be fully configurable.

#### Emulating Dynamically

Two approaches were employed to solve the extensive computational requirement for simulating large networks. First, all neurons were not physically implemented on silicon as it was unnecessary, and second, time-multiplexing was used to leverage the high-speed of the FPGA (Cassidy et al., 2011; Wang et al., 2014a,b, 2017). A single physical minicolumn (100 physical neurons in parallel) could be time-multiplexed to simulate 200 k time-multiplexed (TM) minicolumns, each one updated every millisecond. Limited by the hardware resources (mainly the memory), the cortex emulator was designed to be capable of simulating up to 200 k TM minicolumns in real time and 1 M (2^20^) TM minicolumns at five times slower than real time, i.e., an update every 5 ms.

#### Hierarchical Communication

The presented cortex emulator uses a hierarchical communication scheme such that the communication cost between the neurons can be reduced by orders of magnitude. Anatomical studies of the cortex presented in Thomson and Bannister (2003) showed that cortical neurons are not randomly wired together and that the connections are quite structural. The connection types of the neurons, the minicolumns, and the hypercolumns were stored in a hierarchical fashion instead of individual point-to-point connections. In this scheme, the addresses of the events consist of hypercolumn addresses and minicolumn addresses. Both are generated on the fly with connection parameters according to their connection levels, respectively. This method only requires several kilobytes of memory and can be easily implemented with on-chip SRAMs.

Inspired by observations from neurobiology, the communication between the neurons uses events (spike counts) instead of individual spikes. This arrangement models a cluster of synapses formed by an axon onto the dendritic branches of nearby neurons. The neurons of one type within a minicolumn all receive the same events, which are the numbers of the spikes generated by one type of neuron in the source minicolumns within a time step. One minicolumn has up to eight types of neurons, and each type can be connected to any type of neuron in the destination minicolumns. Every source minicolumn was restricted to have the same number of connections to all of the other minicolumns within the same hypercolumn, but these could have different synaptic weights. The primary advantage of using this scheme is that it overcomes a key communication bottleneck that limits scalability for large-scale spiking neural network simulations.

This system allows the events generated by one minicolumn to be propagated to up to 16 hypercolumns, each of which has up to 128 minicolumns, i.e., to 16 × 128 × 100 = 200 k neurons. Each of these 16 connections has a configurable fixed axonal delay (from 1 to 16 ms, with a 1 ms step).

### Hardware Implementation

The cortex emulator was deliberately designed to be scalable and flexible, such that the same architecture could be implemented either on a standalone FPGA board or on multiple parallel FPGA boards.

**Figure 6:**
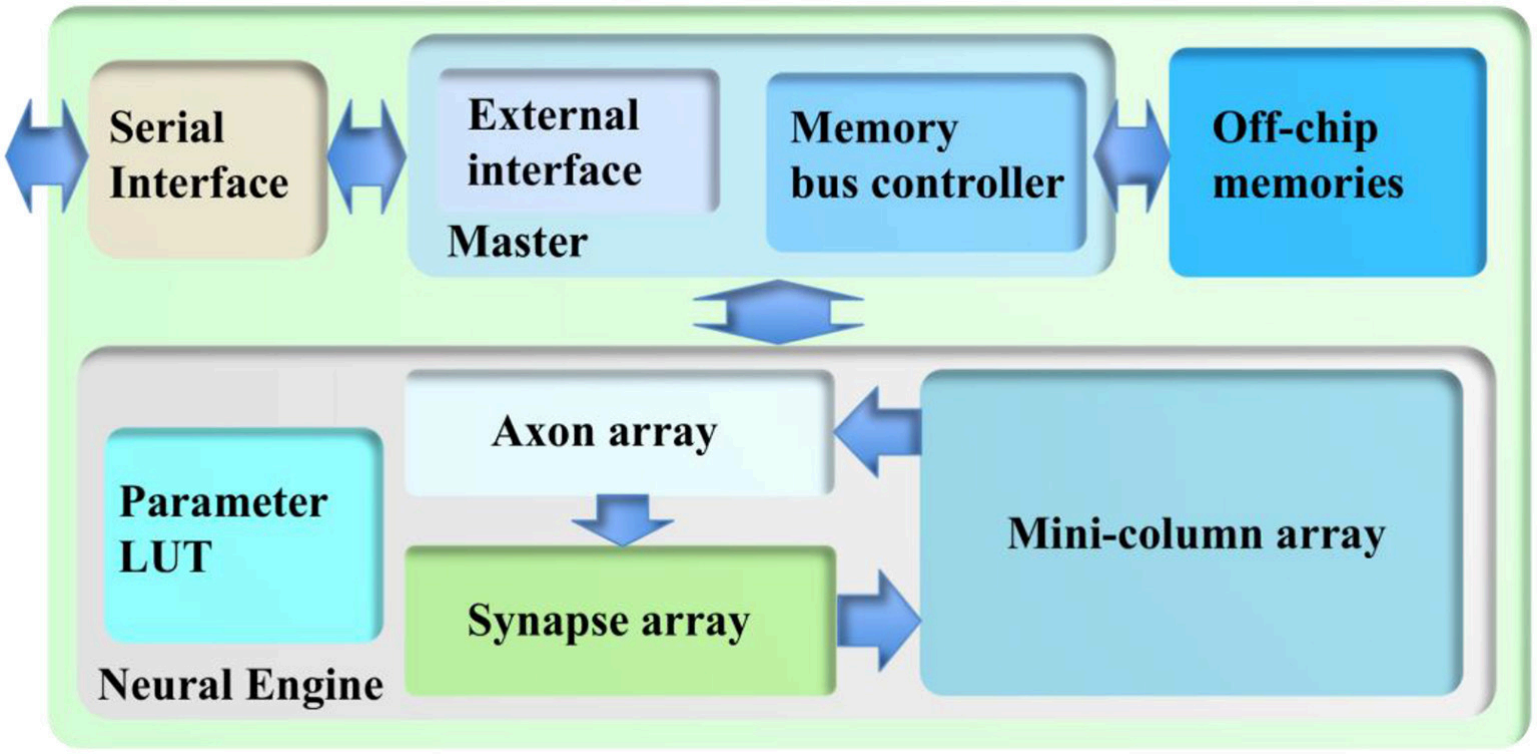
The architecture of the cortex emulator. The system consists of a neural engine, a Master, off-chip memories, and a serial interface. The neural engine realizes the function of biological neural systems by emulating their structures. The Master controls the communication between the neural engine and the off-chip memories, which store the neural states and the events. The serial interface is used to interact with the other FPGAs and the host controller, e.g., PCs.

As a proof-of-concept, this architecture is implemented on a Terasic DE5 kit (with one Altera Stratix V FPGA, two DDR3 memories, and four QDRII memories) as a standalone system. Figure 6 shows its architecture, consisting of a neural engine, a Master, off-chip memories, and a serial interface.

The neural engine forms the main body of the system. It contains three functional modules: a minicolumn array, a synapse array, and an axon array. The minicolumn array implements TM minicolumns. The axon array propagates the events generated by the minicolumns with axonal delays to the synapse array. In the synapse array, these events are modulated with synaptic weights and assigned their destination minicolumn address. The synapse array sends these events to the destination minicolumn array in an event-driven fashion. Besides these functional modules, there is a parameter LUT, which stores the neuron parameters, connection types, and connection parameters. The details are presented in the following section.

Because of the complexity of the system and large number of the events, each module in the neural engine was designed to be a slave module, such that a single Master has full control of the emulation progress. The Master has a memory bus controller that controls the access of the external memories. Because time-multiplexing is used to implement the minicolumn array, the neural state variables of each TM neuron (such as their membrane potentials) need to be stored. These are too big to be stored in on-chip memory and have to be stored in off-chip memory, such as the DDR memory. Using off-chip memory needs flow control for the memory interface, which makes the architecture of the system significantly more complex, especially if there are multiple off-chip memories. The axon array also needs to access the off-chip memories for storing events.

The Master also has an external interface module that performs flow control for external input and output. This module also takes care of instruction decoding. The serial interface is a high-speed interface, such as the PCIe interface, that communicates with the other FPGAs and the host PC. It is board-dependent, and Altera's 10 G base Phy IP is used here.

#### Minicolumn Array

**Figure 7:**
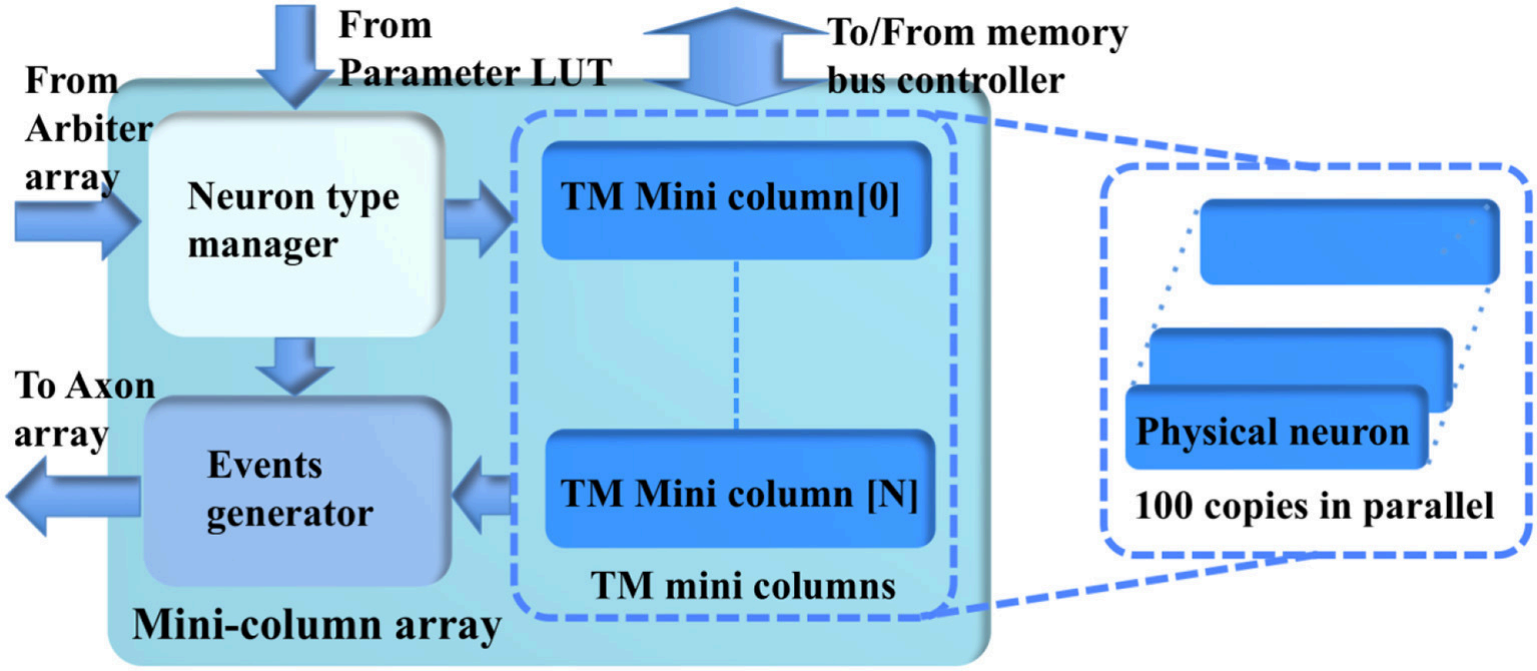
The structure of the minicolumn array.

The minicolumn array (Figure 7) consists of a neuron-type manager, an event generator, and the TM minicolumns, which have 100 parallel physical neurons. These neurons generate positive (excitatory) and negative (inhibitory) post-synaptic currents (EPSCs and IPSCs) from input events weighted in the synapse array. These PSCs are integrated in the cell body (the soma). The soma performs a leaky integration of the PSCs to calculate the membrane potential and generates an output spike (post-synaptic spike) when the membrane potential passes a threshold, after which the membrane potential is reset and enters a refractory period. Events, i.e., spike counts, are sent to the axon array together with the addresses of the originating minicolumns, the number of connections, and axonal delay values for each connection (between two minicolumns).

#### Axon Array

The axon array propagates the events from the minicolumn array or from the external interface to the synapse array, using programmable axonal delays. To implement this function on hardware, a two-phase scheme comprising a TX-phase and an RX-phase is used. In the TX-phase, the events are written into different regions of the DDR memories according to their programmable axonal delay values. In the RX-phase, for each desired axonal delay value, the events are read out from the corresponding region of the DDR memories stochastically, such that their expected delay values are approximately equal to the desired ones.

#### Synapse Array

The synapse array emulates the function of biological synaptic connections: it modulates the incoming events from the axon array with synaptic weights and generates destination minicolumn addresses for them. These events are then sent to the TM minicolumns. The synapse array only performs the linear accumulation of synaptic weights of the incoming events, whereas the exponential decay is emulated by the PSC generator in the neuron.

#### Master

The Master plays a vital role in the cortex emulator: it has complete control over all modules in the neural engine such that it can manage the progress of the simulation. This mechanism effectively guarantees no event loss or deadlock during the simulation. The Master slows down the simulation by pausing the modules that are running quicker than other modules. The Master has two components (Figure 6): a memory bus controller and an external interface. The memory bus controller has two functions: (i) interfacing the off-chip memory with Altera IPs, and (ii) managing the memory bus sharing between the minicolumn array and the axon array.

The external interface module controls the flow of the input and output of events and parameters. The main input to this system is events, which are sent to the minicolumn array via the axon array. This module also performs instruction decoding such that the parameter LUT and the system registers can be configured. The outputs of the emulator are individual spikes (100 bits, one per neuron) and events generated by the minicolumns.

### Programming API

Along with the hardware platform, a simple application programming interface (API) was developed in Python that is similar to the PyNN programming interface (Davison et al., 2008). This API is very similar to the high-level object-oriented interface that has been defined in the PyNN specification: it allows users to specify the parameters of neurons and connections, as well as the network structure using Python. This will enable the rapid modeling of different topologies and configurations using the cortex emulator. This API allows monitoring of the generated spikes in different hypercolumns. As future work, the plan is to provide full support for PyNN scripts and incorporate interactive visualization features on the cortex emulator.

### Application

This emulator will be useful for computational neuroscientists to run large-scale spiking neural networks with millions of neurons in real time. In one of the applications, it is being used to emulate the auditory cortex in real time (Wang et al., 2018).

## BrainScaleS

The BrainScaleS neuromorphic system has been developed at the University of Heidelberg in collaboration with the Technical University Dresden and the Fraunhofer IZM in Berlin. The BrainScaleS neuromorphic system is based on the direct emulation of model equations describing the temporal evolution of neuron and synapse variables. The electronic neuron and synapse circuits act as physical models for these equations. Their measurable electrical quantities represent the variables of the model equations, thereby implicitly solving the related differential equations.

The current first generation BrainScaleS system implements the Adaptive Exponential Integrate-and-Fire Model (Gerstner and Brette, 2009). All parameters are linearly scaled to match the operating conditions of the electronic circuits. The membrane voltage range between hyperpolarization, i.e., the reset voltage in the model, and depolarization (firing threshold) is approximately 500 mV. Time, being a model variable as well, can also be scaled in a physical model. In BrainScaleS, this is used to accelerate the model in comparison to biological wall time. It uses a target acceleration factor of 10^4^. This acceleration factor has a strong influence on most of the design decisions, since the communication rate between the neurons scales directly with the acceleration factor, i.e., all firing rates are also 10^4^ higher than those in biological systems. The rationale behind the whole communication scheme within the BrainScaleS system is based on these high effective firing rates.

**Figure 8:**
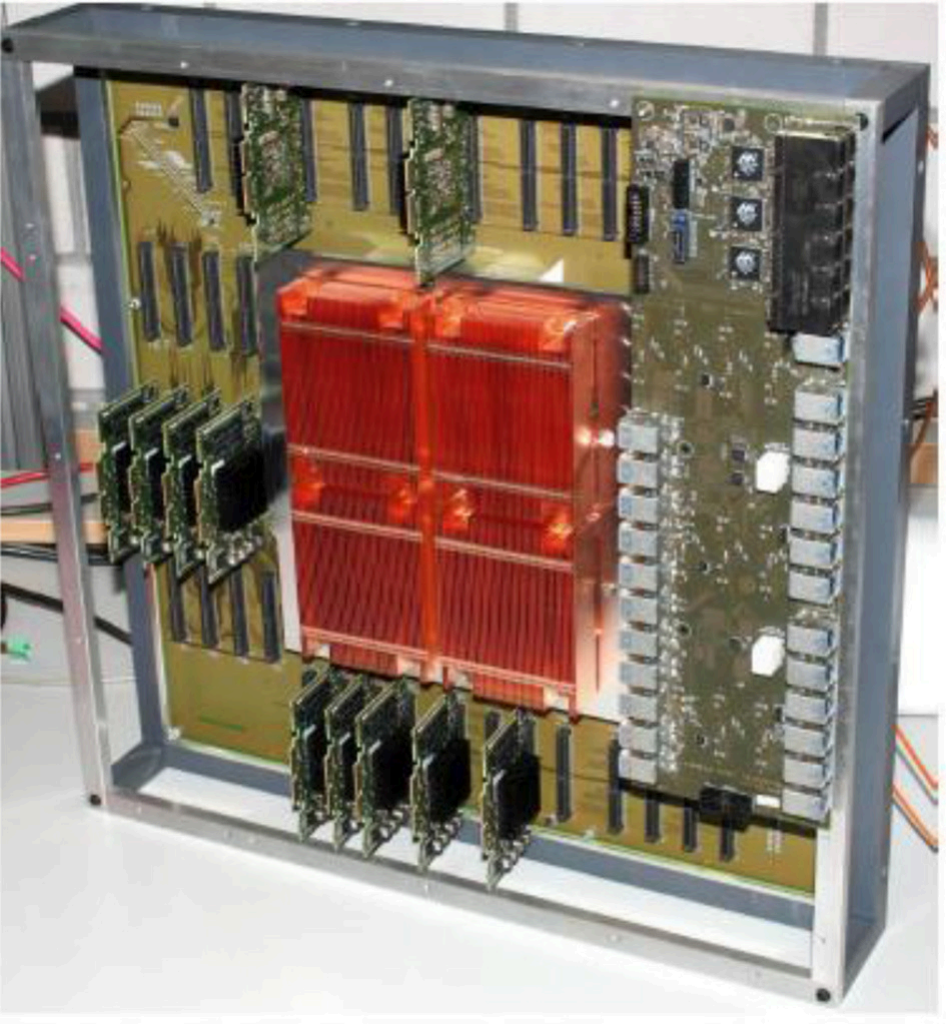
Photograph of a BrainScaleS wafer module with part of the communication boards removed to show the main PCB. The wafer is located beneath the copper heat sink visible in the center.

In the BrainScaleS system, the whole neuron, including all its synapses, is implemented as a continuous-time analog circuit. Therefore, it consumes a substantial silicon area. To be able to implement networks larger than a few hundred neurons, a multichip implementation is necessary. Due to the high acceleration factor, this requires very high communication bandwidth between the individual Application Specific Integrated Circuits (ASICs) of such a multichip system. BrainScaleS uses wafer-scale integration to solve this problem. Figure 8 shows a photograph of a BrainScaleS wafer module integrating an uncut silicon wafer with 384 neuromorphic chips. Its neuromorphic components will be described in the remainder of this section, which is organized as follows: sections HICANN ASIC Explains the Basic Neural Network Circuits, and Communication Infrastructure details the wafer-scale communication infrastructure. Finally, the wafer-scale integration is described in section Wafer Module.

### HICANN ASIC

**Figure 9:**
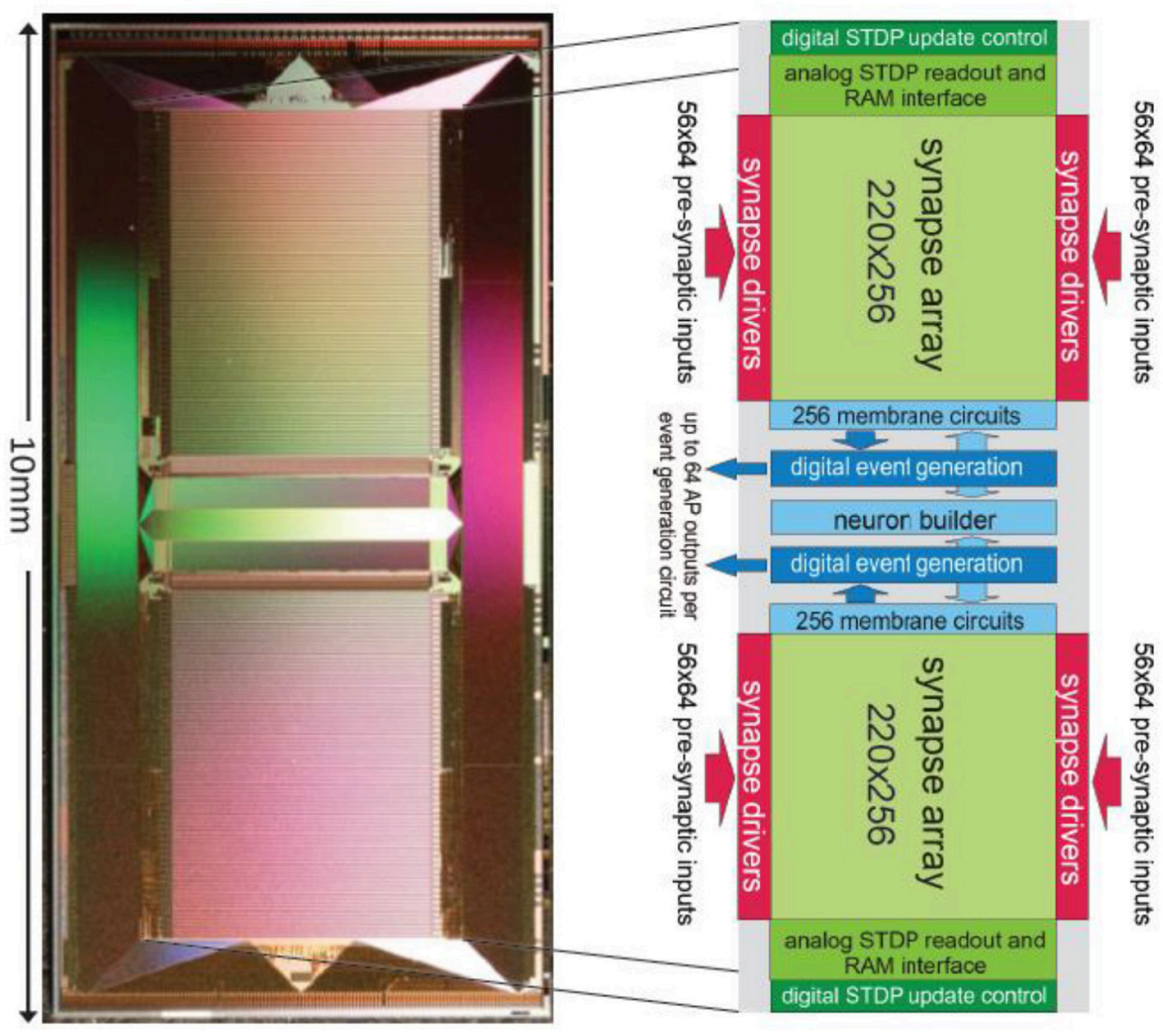
Photograph of a single HICANN die. The enlargement shows a block diagram of the analog network core located in the center of the die.

At the center of the BrainScaleS neuromorphic hardware system is the High-Input Count Analog Neuronal Network Chip (HICANN) ASIC. Figure 9 shows a micro-photograph of a single HICANN die. The center section contains the symmetrical analog network core: two arrays with synapse circuits enclose the neuron blocks in the center. Each neuron block has an associated analog parameter storage. The communication network surrounding the analog network core is described in section Communication Infrastructure. The first HICANN prototype is described in Schemmel et al. (2010). The current BrainScaleS system is built upon the third version of the HICANN chip, which is mostly a bug-fix version. A second-generation BrainScaleS system based on a smaller manufacturing process feature size is currently under development. It shrinks the design geometries from 180 to 65 nm and improves part of the neuron circuit (Aamir et al., 2016), adds hybrid plasticity (Friedmann et al., 2016), and integrates a high-speed Analog-to-Digital Converter (ADC) for membrane voltage readout. This paper refers to the latest version of the first generation HICANN chip as it is currently used in the BrainScaleS system.

**Figure 10:**
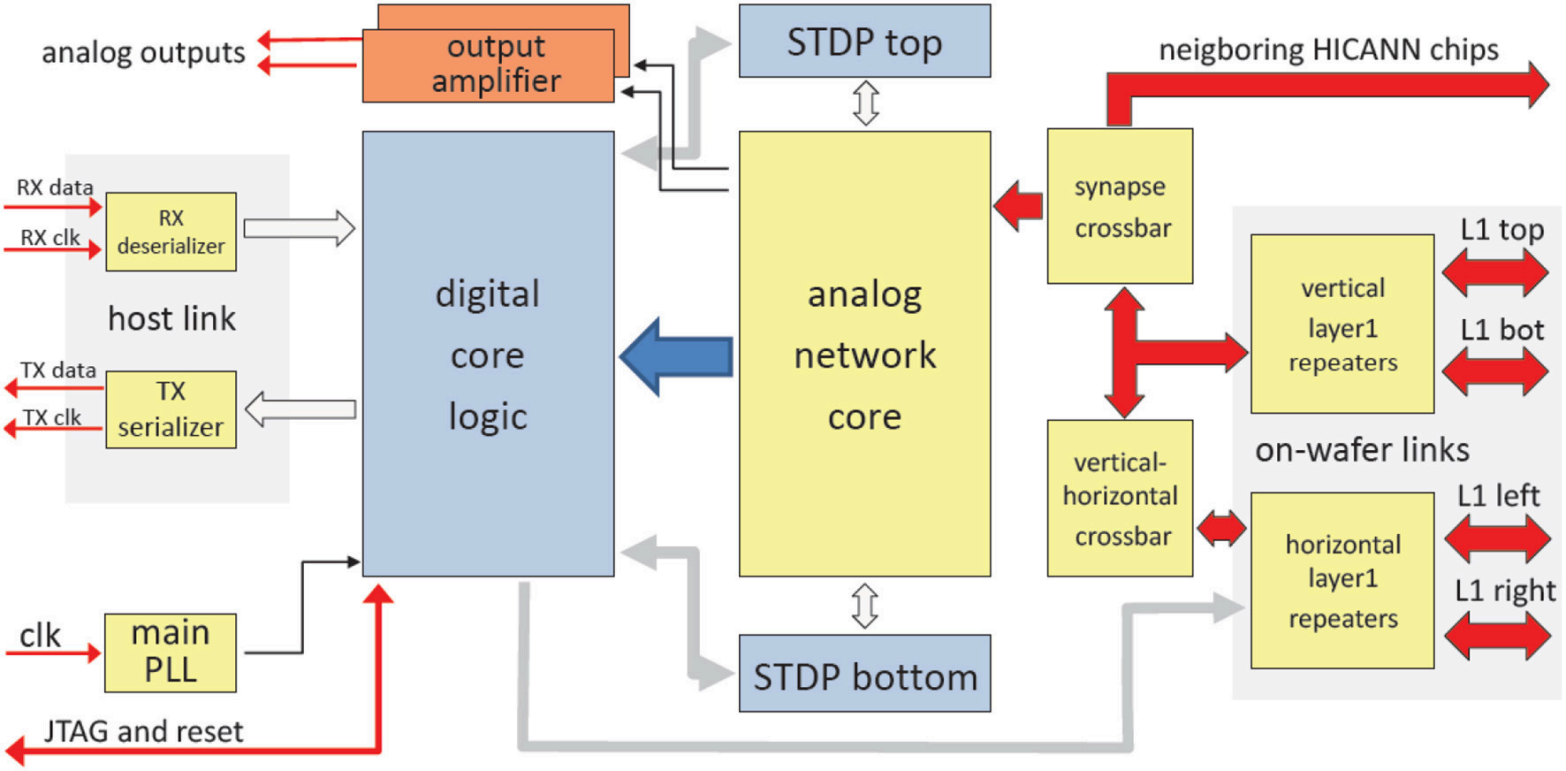
Block diagram of the HICANN chip.

Figure 10 shows the main components of the HICANN chip. The analog network core contains the actual analog neuron and synapse circuits. The network core communicates by generating and receiving digital event signals, which correspond to biological action potentials. One major goal of HICANN is a fan-in per neuron of more than 10 k pre-synaptic neurons. To limit the input ports of the analog core to a manageable number, time-multiplexing is used for event communication: each communication channel is shared by 64 neurons. Each neuronal event therefore transmits a 6-bit number while time is coded by itself, i.e., event communication happens in real-time related to the emulated network model. This communication model is subsequently called Layer 1 (L1).

Layer 2 (L2) encoding is used to communicate neural events in-between the wafer and the host compute cluster. Due to the latencies involved with long-range communication, it is not feasible to use a real-time communication scheme. A latency of 100 ns would translate to a 1 ms delay in biological wall time using an acceleration factor of 10^4^. Therefore, a protocol based on packet-switching and embedded digitized time information is used for the L2 host communication.

The gray areas labeled “digital core logic” and “STDP top/bottom” are based on synthesized standard-cell logic. These are not visible in Figure 9, because the L1 communication lines are located on top of the standard cell areas (section Communication Infrastructure). They occupy the whole area surrounding the analog network core. The core itself uses a full-custom mixed-signal implementation, as do the repeaters, Serializer/De-Serializer (SERDES) and Phase-Locked Loop (PLL) circuits. The only purely analog components are two output amplifiers. They allow direct monitoring of two selectable analog signals.

The RX- and TX-circuits implement a full-duplex high-speed serial link to communicate with the wafer module. The thick red arrows represent the physical L1 lanes. Together with the vertical repeater circuits and the synapse and vertical-horizontal crossbars, they form the L1 network (Section Communication Infrastructure).

#### Neuron Circuits

**Figure 11:**
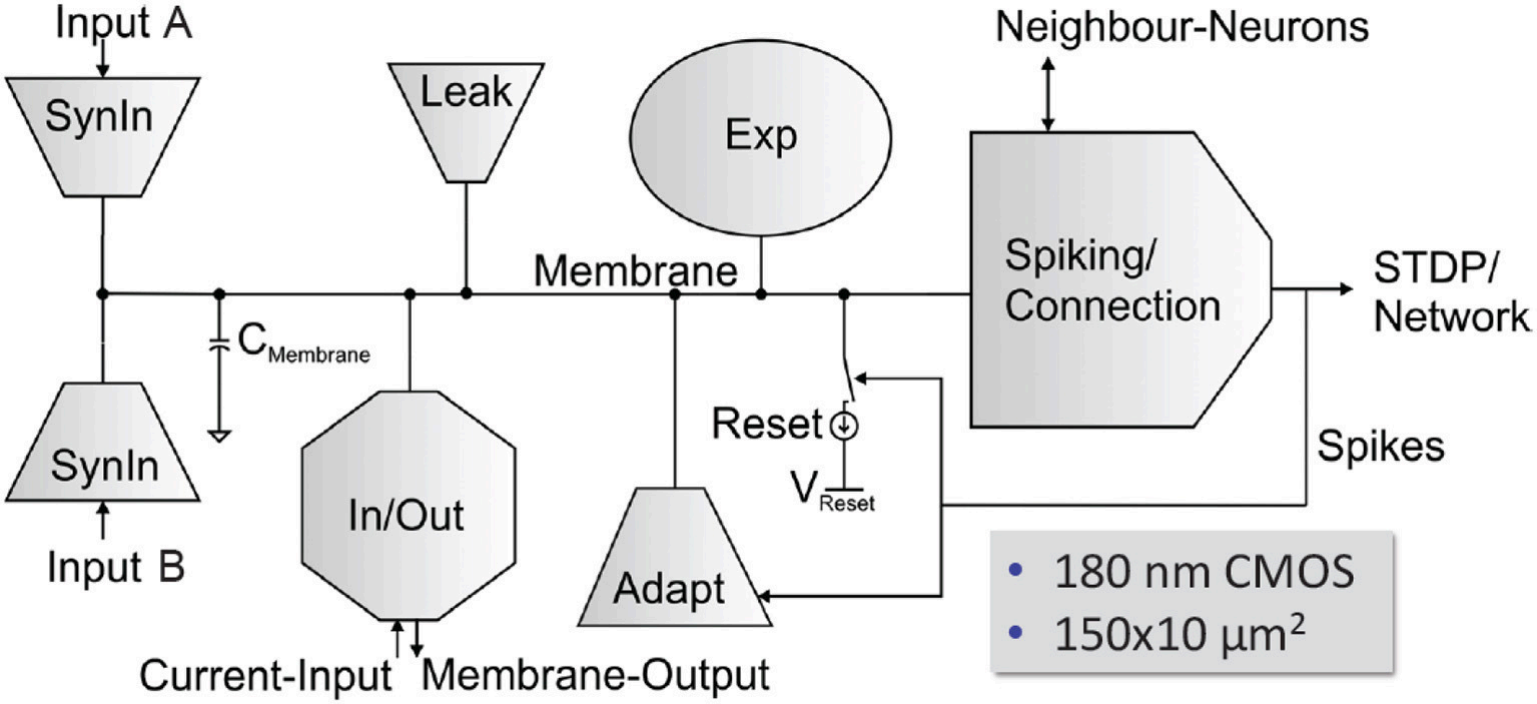
Block diagram of a HICANN membrane circuit. The model neurons are formed by interconnecting groups of membrane circuits.

The HICANN neuron circuits are based on the AdEx model (Naud et al., 2008). Details of the circuit implementation of this model and measurement results of the silicon neuron can be found in Millner et al. (2010) and Millner (2012). Figure 11 shows the basic elements of a membrane circuit. A row of 256 membrane circuits is located adjacent to each of the two synapse arrays. Within the two-dimensional synapse arrays, each membrane circuit has one column of 220 synapses associated with it. To allow for neurons with more than 220 inputs, up to 64 membrane circuits can be combined to one effective neuron.

The circuit named “neuron builder” in Figure 9 is responsible for interconnecting the membrane capacitances of the membrane circuits that will operate together as one model neuron. Each membrane circuit contains a block called “Spiking/Connection,” which generates a spike if the membrane crosses its threshold voltage. This spike generation circuit can be individually enabled for each membrane circuit. In neurons built by interconnecting a multitude of membrane circuits, only one spike generation circuit is enabled. The output of the spike generation circuit is a digital pulse lasting for a few nanoseconds. It is fed into the digital event generation circuit located below each neuron row as shown in Figure 9.

Additionally, each membrane circuit sends the spike signal back into its synapse array column, where it is used as the post-synaptic signal in the temporal correlation measurement between pre- and post-synaptic events. Within a group of connected membrane circuits, their spike signals are connected as well. Thereby, the spike signal from the single enabled spike generation circuit is reflected in all connected membrane circuits and driven as post-synaptic signal in all synaptic columns belonging to the connected membrane circuits. The neuron uses 23 analog parameters for calibration. They are split in 12 voltage parameters, like the reversal potentials or the threshold voltage of the neuron, and 11 bias currents. These parameters are stored adjacent to the neurons in an array of analog memory cells. The memory cells are implemented using single-poly floating-gate (FG) technology.

Each membrane circuit is connected to 220 synapses by means of two individual synaptic input circuits. Each row of synapses can be configured to use either of them, but not both simultaneously. Usually they are configured to model the excitatory and inhibitory inputs of the neuron.

**Figure 12:**
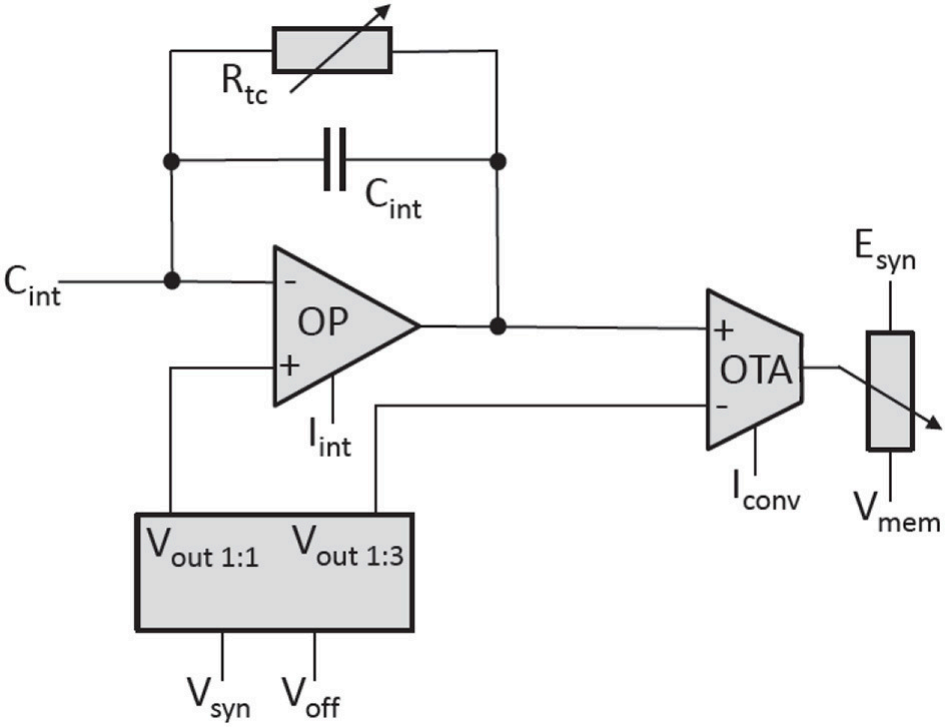
Simplified circuit diagram of the synaptic input of the neuron.

The synaptic input uses a current-mode implementation. An operational amplifier (OP) acts as an integrator located at the input (see Figure 12). It keeps the voltage level of the input constant at *V*_*syn*_. Each time a synapse receives a pre-synaptic signal, it sinks a certain amount of current for a fixed time interval of nominal 4 ns. To restore the input voltage level to *V*_*syn*_, the integrator must source the corresponding amount of charge through its feedback capacitor.

The second amplifier is an Operational Transconductance Amplifier (OTA) that converts the voltage over the feedback capacitor *C*_*int*_ into a current that is subsequently used to control a current-controlled resistor connecting the reversal potential to the membrane. The exponential decay of the synaptic conductance is generated by the adjustable resistor *R*_*tc*_ in parallel to *C*_*int*_. The rise-time of the synaptic conductance is controlled by the bias current *I*_*int*_ of the integrator. The bias current of the OTA, *I*_*conv*_, sets the ratio between the conductance and the synaptic current.

#### Synapse Array and Drivers

**Figure 13:**
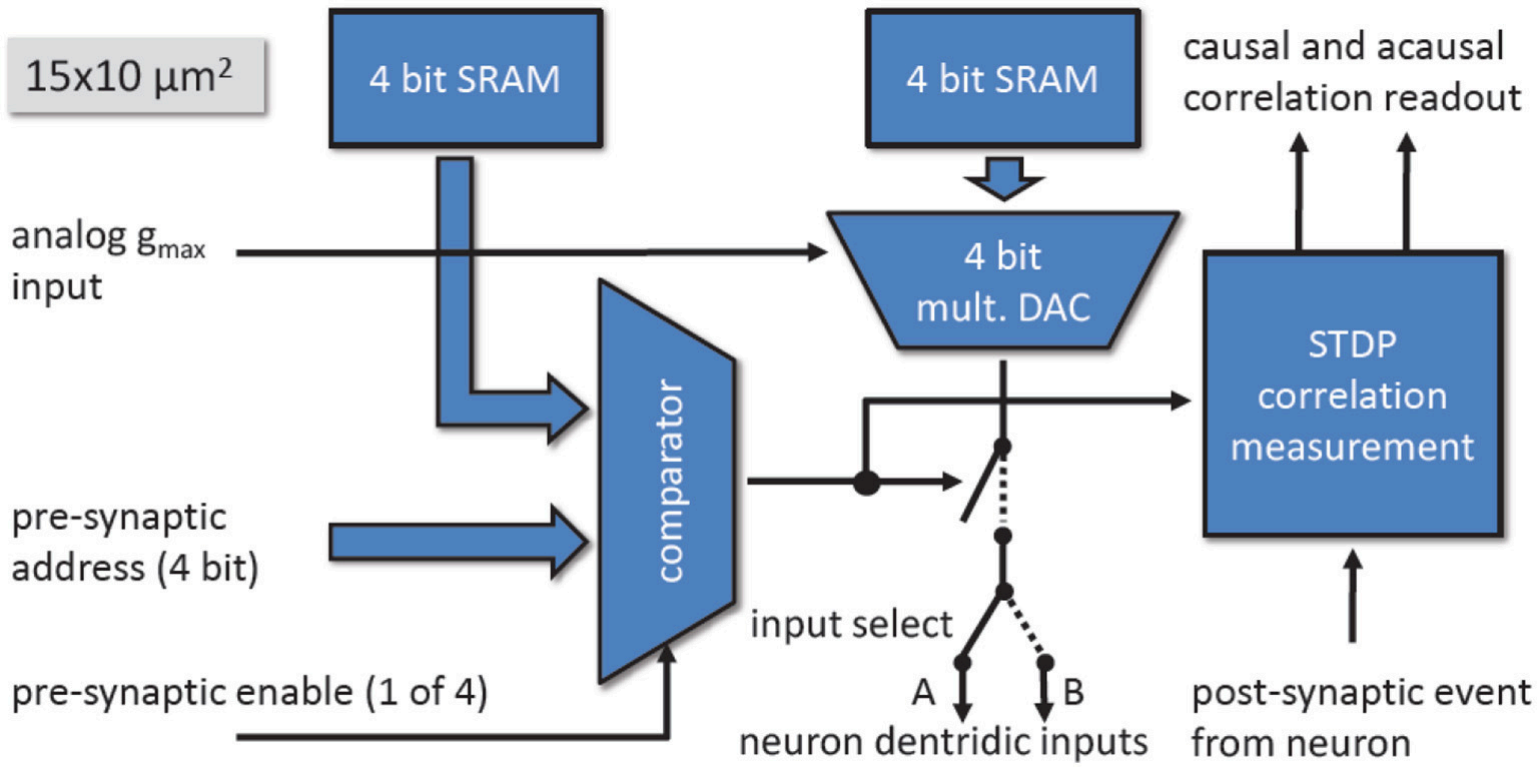
Block diagram of the synapse.

Figure 13 shows the different components of the synapse. The control signals from the synapse drivers run horizontally through the synapse array, orthogonal to the neuron dendritic current inputs (synaptic input). The input select signal statically determines which current input of the membrane circuits the synapses use. It can be set individually for each row of synapses.

A synapse is selected when the 4-bit pre-synaptic address matches the 4-bit address stored in the address memory while the pre-synaptic enable signal is active. The synaptic weight of the synapse is realized by a 4-bit Digital-to-Analog Converter (DAC) and a 4-bit weight memory. The output current of the DAC can be scaled with the row-wise bias *g*_*max*_. During the active period of the pre-synaptic enable signal, the synapse sinks the current set by the weight and *g*_*max*_.

By modulating the length of the pre-synaptic enable signal, the total charge sunk by the synapse can be adjusted for each pre-synaptic spike. The synapse drivers use this to implement short-time plasticity.

Each synapse contains in addition to the current sink, a correlation measurement circuit to implement Spike Timing Dependent Plasticity (STDP) (Friedmann, 2009). The pre-synaptic signals are generated within the synapse drivers, located to the left and right side of the synapse array in Figure 9. Each synapse driver controls two adjacent rows of synapses.

### Communication Infrastructure

The analog network core described in the previous section implements a total of 220 synapse driver circuits. Each synapse driver receives one L1 signal.

The physical L1 connections use the topmost metal layer (metal 6) for vertical lines and the metal layer beneath for horizontal lines. Since the synapse array uses all metal layers, the only physical space for L1 connections in HICANN is the area surrounding the analog network core and the center of the analog network core, above the digital event generation and neuron builder circuits shown in Figure 10.

There are 64 horizontal lines in the center and 128 to the left and to the right of the analog network core. Due to the differential signaling scheme used, each line needs two wires; the total number of wires used is 640, each wire pair using a maximum signal bandwidth of 2 Gbits^−1^. This adds up to a total bandwidth of 640 Gbits^−1^.

#### Wafer-Scale Integration

To increase the size of an emulated network beyond the number of neurons and synapses of a single HICANN chip, chip-to-chip interconnect is necessary. Any 3D integration technique (Ko and Chen, 2010) will allow to stack only a few chips reliably. Therefore, on its own, 3D stacking is not the solution to scale HICANN to large network sizes.

Packaging the HICANN chips for flip-chip PCB mounting and soldering them to carrier boards would be a well-established option. Driving the additional capacity and inductance of two packages and a PCB trace would make the simple asynchronous differential transmission scheme unfeasible. Therefore, the area and power used by the communication circuits would most likely increase. To solve the interconnection and packaging problems a different solution was chosen: wafer-scale integration. Wafer-scale integration, i.e., the usage of a whole production wafer instead of dicing it into individual reticles, is usually not supported by the semiconductor manufacturing processes available for university projects. Everything is optimized for mass-market production, where chip sizes rarely reach the reticle limit. Stitching individual reticles together on the top metal layer was therefore not available for HICANN. In the 180 nm manufacturing process used, eight HICANN dies fit on one reticle. So, two problems had to be solved: how to interconnect the individual reticles on the wafer and how to connect the whole wafer to the system.

Within a reticle, the connections between the L1 lines of neighboring HICANN chips are made directly by top layer metal. With stitching available, interconnects on top layer metal would allow to continue the L1 lines across reticle boundaries. But, the problem of the wafer to PCB connection would remain. The solution employed in BrainScaleS solves both connection problems and can be used with any silicon wafer manufactured in a standard CMOS process. It is based on the post-processing of the manufactured wafers. A multi-layer wafer-scale metalization scheme has been developed by the Fraunhofer IZM in Berlin. It uses a wafer-scale maskset with μm resolution.

**Figure 14:**
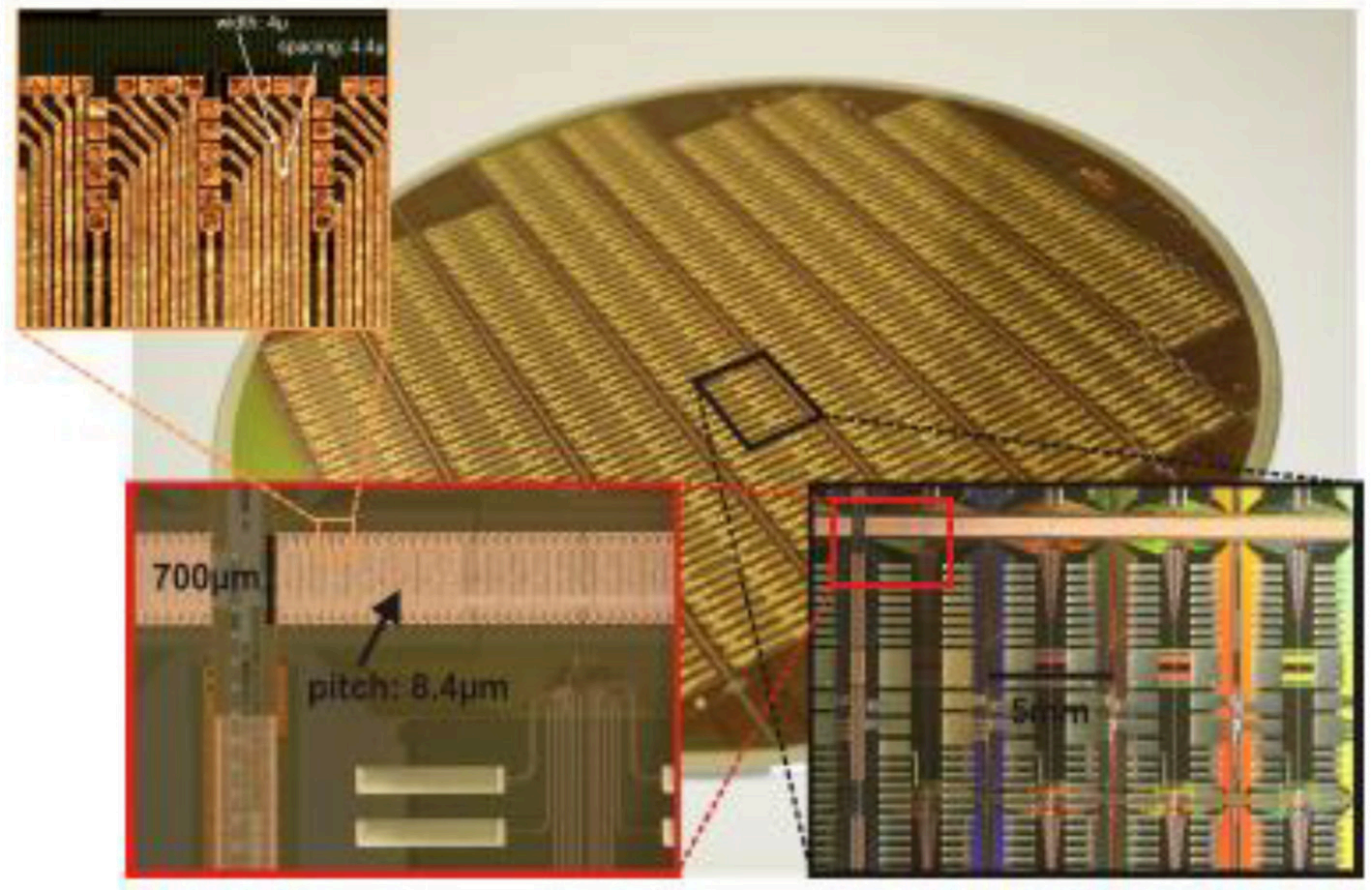
L1 post processing. Main picture: Photograph of a post-processed wafer. Enlargements: bottom right: top-left corner of a reticle. The large stripes connect the wafer to the main PCB. Bottom left: the L1 connections crossing the reticle border. Top left: 4-μm wide individual metal traces of the L1 connections terminating at the pad-windows of the top reticle.

After some initial research, a minimum pad window size of 5 × 5 μm^2^ was chosen. 15 × 15 μm^2^ copper areas have proven to connect reliably to these pad windows. In Figure 14, a photograph with all post-processing layers applied to a wafer of HICANN chips is shown. The cut-out with the maximum enlargement in the top left corner shows the dense interconnects linking the L1 lines of two HICANN chips in adjacent reticles. They use a pitch of 8.4 μm^1^. Due to the larger size of the post-processing to wafer contacts, some staggering is necessary.

A second, much thicker post-processing layer is used to create the large pads visible in the eight columns in the inner area of the reticle. They solve the second problem related to wafer-scale integration: the wafer to PCB connection. Since the L1 connections are only needed at the edge of the reticle, the whole inner area is free to redistribute the HICANN IO and power lines and connect them to the regular contact stripes visible in the photograph.

### Wafer Module

A photograph of a partial assembled wafer module is shown in Figure 9. The visible components are the main PCB with the wafer in the center, beneath the copper heat-sink. There are 48 connectors for the Communication Subgroup (CS) surrounding the wafer. Each CS provides the Low-Voltage Differential Signaling (LVDS) and Joint Test Action Group (JTAG) signals for one reticle^2^. Each wafer module uses 481 Gbit/s Ethernet links to communicate with the host compute cluster via an industrial standard Ethernet switch hierarchy.

The links are provided by four IO boards mounted on top of the CSs. In Figure 9, only one of them is in place to avoid blocking the view on the CSs. Twelve RJ45 Ethernet Connectors (RJ45s) can be seen in the upper right corner of the IO board. The remaining connectors visible are for future direct wafer-to-wafer networking.

#### Wafer-PCB Connection

As shown in Figure 14, the HICANN wafer is connected to the main PCB by a contact array formed by the wafer post-processing. The stripes visible in the figure have a width of 1.2 mm and a pitch of 400 μm. A mirror image of theses stripes is placed on the main PCB.

The connection between both stripe patterns is then formed by Elastomeric Connectors^3^ with a width of 1 mm and a density of five conducting stripes per mm. Therefore, they do not have to be precisely aligned to the PCB or the wafer, only the wafer and the PCB have to be aligned to each other with about 50 μm accuracy. This is achieved by placing the wafer in an aluminum bracket, which is fastened to an aluminum frame located on the opposite side of the main PCB by eight precision screws.

To place the Elastomic Connectors at the correct positions, they are held by a thin, slotted FR4 mask which is screwed to the main PCB. A special alignment tool has been developed to optically inspect and correct the wafer-to-PCB alignment during the fastening of the screws. The optimum pressure is achieved by monitoring the electrical resistance of a selected set of wafer-to-PCB contacts during the alignment and fastening process. After the wafer is correctly in place, the bracket is filled with nitrogen and sealed.

### Application

By compressing the model timescale by several orders of magnitude, the system allows to model processes like learning and development in seconds instead of hours. It will make parameter searches and statistical analysis possible in all kind of models. Some results of accelerated analog neuromorphic hardware are reported in Petrovici et al. (2016, 2017a,b) and Schmitt et al. (2016).

## Dynap-SEL: A Multi-Core Spiking Chip for Models of Cortical Computation

Novel mixed-signal multi-core neuromorphic processors that combine the advantages of analog computation and digital asynchronous communication and routing have been recently designed and fabricated in both standard 0.18 μm CMOS processes (Moradi et al., 2018) and advanced 28 nm Fully-Depleted Silicon on Insulator (FDSOI) processes (Qiao and Indiveri, 2016) in the lab of Giacomo Indiveri at the University of Zurich, Switzerland. The analog circuits used to implement neural processing functions have been presented and characterized in Chicca et al. (2014). Here, the routing and communication architecture of the 28 nm “Dynamic Neuromorphic Asynchronous Processor with Scalable and Learning” (Dynap-SEL) device is described, highlighting both its run-time network re-configurability properties and its on-line learning features.

### The Dynap-SEL Neuromorphic Processor

The Dynap-SEL chip is a mixed-signal multi-core neuromorphic processor that comprises four neural processing cores, each with 16 × 16 analog neurons and 64 4-bit programmable synapses per neuron, and a fifth core with 1 × 64 analog neuron circuits, 64 × 128 plastic synapses with on-chip learning circuits, and 64 × 64 programmable synapses. All synaptic inputs in all cores are triggered by incoming Address Events (AEs), which are routed among cores and across chips by asynchronous Address-Event Representation (AER) digital router circuits. Neurons integrate synaptic input currents and eventually produce output spikes, which are translated into AEs and routed to the desired destination via the AER routing circuits.

#### Dynap-SEL Routing Architecture

**Figure 15:**
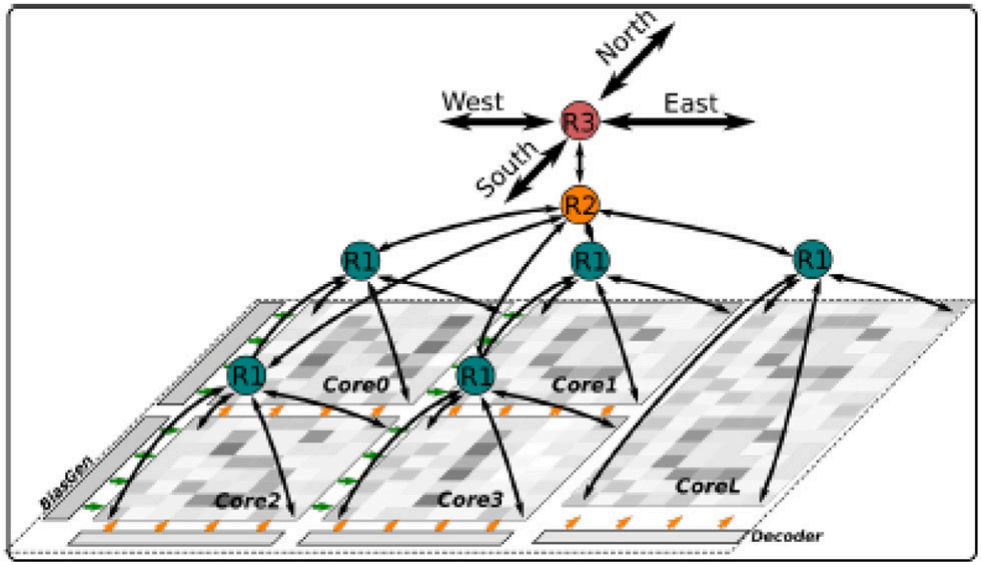
Dynap-SEL with hierarchical routers. Each Dynap-SEL comprises four TCAM-based non-plastic cores and one plastic core. The hierarchical routing scheme is implemented on three levels for intra-core (R1), inter-core (R2), and inter-chip (R3) communication.

The Dynap-SEL routing architecture is shown in Figure 15. It is composed of a hierarchy of routers at three different levels that use both source-address and destination-address routing. The memory structures distributed within the architecture to support the heterogeneous routing methods employ both Static Random Access Memory (SRAM) and Ternary Content Addressable Memory (TCAM) memory elements (see Moradi et al., 2018 for a detailed description of the routing schemes adopted). To minimize memory requirements, latency, and bandwidth usage, the routers follow a mixed-mode approach that combines the advantages of mesh routing (low bandwidth usage, but high latency), with those of hierarchical routing (low latency, but high bandwidth usage) (Benjamin et al., 2014). The asynchronous digital circuits in Dynap-SEL route spikes among neurons both within a core, across cores, and across chip boundaries. Output events generated by the neurons can be routed to the same core, via a Level-1 router R1; to other cores on the same chip, via a Level-2 router R2; or to cores on different chips, via a Level-3 router R3. The R1 routers use source-address routing to broadcast the address of the sender node to the whole core and rely on the TCAM cells programmed with appropriate tags to accept and receive the AE being transmitted. The R2 routers use absolute destination-address routing in a 2D tree to target the desired destination core address. The R3 routers use relative destination address-routing to target a destination chip at position (Δ*x*, Δ*y*). The memory used by the routers to store post-synaptic destination addresses is implemented using 8.5 k 16-bit SRAM blocks distributed among the Level-1, -2, and -3 router circuits.

In each non-plastic core, 256 analog neurons and 16 k asynchronous TCAM-based synapses are distributed in a 2D array. Each neuron in this array comprises 64 synapses with programmable weights and source-address tags. Thanks to the features of the TCAM circuits, there are 2^11^ potential input sources per synapse. Synaptic input events are integrated over time by a Differential Pair Integrator (DPI) linear integrator circuit (Bartolozzi and Indiveri, 2007) that exhibits dynamics with biologically realistic time constants (e.g., of the order of tens of milliseconds). Thanks to its modularity, scalability, and on-chip programmable routers, the Dynap-SEL can be integrated in a chip array of up to 16 × 16 chips, allowing all-to-all connectivity among all neurons in the array. This enables the implementation of a wide range of connections schemes, without requiring any additional external mapping, memory, or computing support. By following the parallel AER protocol, it is possible to establish direct communications between Dynap-SEL chips and other AER sensors and computing devices, enabling the construction of large-scale sensory processing systems.

The TCAM and SRAM circuits are subdivided into small memory blocks and embedded within the neuron and synapse arrays. Given that memory and computation are co-localized, each memory access operation is extremely efficient in terms of power consumption, compared to the classical scheme of accessing large TCAM/SRAM blocks placed at longer distances. As there are only local and sparse memory access operations, the requirement of memory bandwidth is also much lower than in the traditional von Neumann architecture. In addition to the power and bandwidth benefits, this distributed heterogeneous memory architecture lends itself well to the exploitation of emerging memory technologies, e.g., by replacing the CMOS Content Addressable Memory (CAM) or SRAM cells with nano-scale Resistive Random Access Memories (ReRAMs) or memristors (Vianello et al., 2014; Chen et al., 2017). By careful design of the analog circuits, an extremely compact layout can be achieved. This allowed the implementation of multiple physical analog neurons for true parallel computing, rather than using time-multiplexing to share the computing resources of digital neural processing blocks (e.g., as it is done in Merolla et al., 2014). In the 28 nm process used, the analog neurons occupy around 5% of whole chip area.

#### The Plastic Core

**Figure 16:**
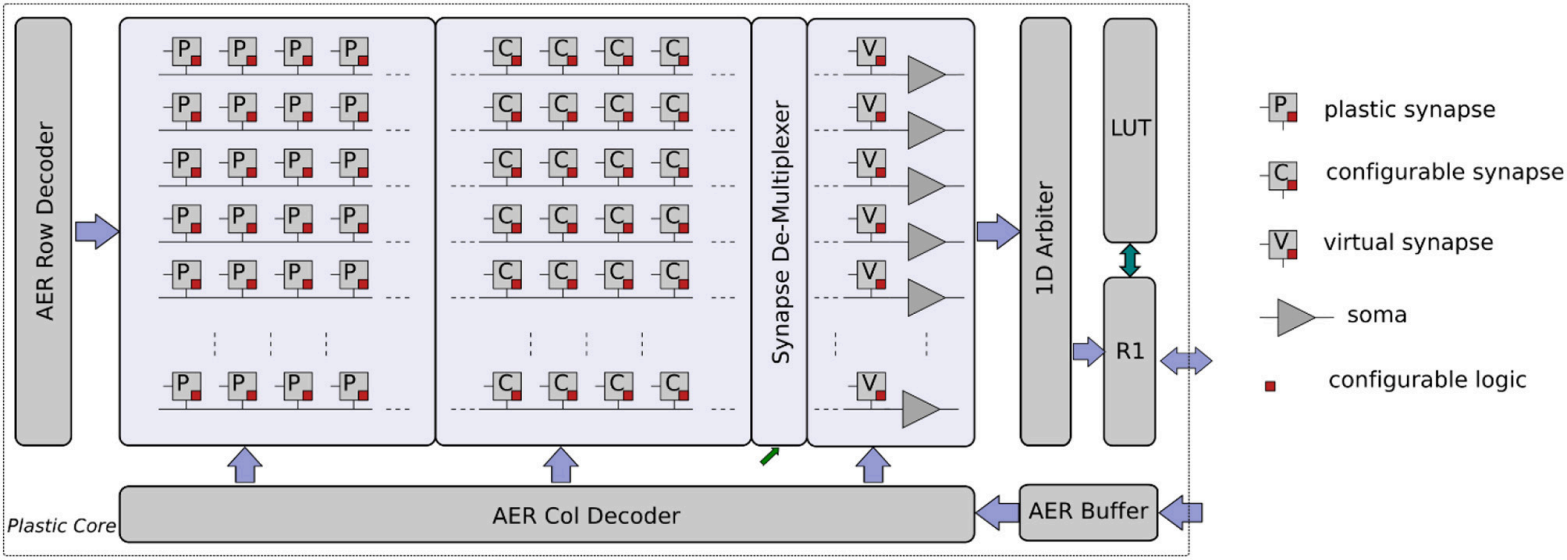
Plastic core architecture. It comprises a 64 × 128 array of plastic synapses, a 64 × 64 array of non-plastic synapses, a 64 × 4 array of time-multiplexed linear synapses, a synapse row de-multiplexer, and 64 adaptive I&F neurons. Each synapse has digital memory and configuration logic circuits. In addition, there are AER input Row/Column decoders, a 1D AER output arbiter, a local router R1, and a corresponding LUT for routing.

This core in the Dynap-SEL device comprises 64 analog neurons. Each neuron has 128 mixed-signal plastic synapses, 64 mixed-signal non-plastic synapses, and 4 linear synapse circuits. The plastic and non-plastic synapses have synaptic weight parameters with 4-bit resolution, while the linear synapse circuits have four independent sets of parameters that can be set by 12-bit programmable bias-generators. These parameters can be used to change the synaptic weights, time constants, or type of excitatory/inhibitory synapse. Furthermore, each of the linear synapses can be time-multiplexed to represent many different synaptic inputs that have the same weight and temporal dynamics (e.g., a 1 KHz input spike train could represent 1,000 1 Hz synaptic inputs). The block diagram of the plastic core is shown in Figure 16.

The weight update mechanism of the plastic synapses is governed by a 4-bit up/down counter, which is used to implement the spike-driven learning rule proposed in Brader et al. (2007) (see also Qiao et al., 2015 for a detailed description of the learning circuits involved). Local digital latches are placed in each synapse to set the synapse type (excitatory/inhibitory), to impose a user-specified weight value, to enable/disable local weight monitor circuits, to enable/disable the learning, or to enable/disable the access to the input broadcast line. Non-plastic synapse circuits are a simplified version of the plastic ones, which do not have the on-line learning mechanism, but share all other features. A local pulse-to-current converter is placed in each synapse to convert the fast AER input events into longer current pulses with tunable pulse width, to provide an additional degree of control over the synaptic weight amplitude range. The long weighted current pulses are then summed and integrated by DPI filter circuits (Bartolozzi and Indiveri, 2007) located at the side of the synapse array. The filtered output of the weighted sum of currents is then fed into their corresponding neuron circuit. A synapse demultiplexer allows the user to assign more synapse rows to targeted neurons, to increase the size of the input space/neuron (at the cost of decreasing the number of active neurons). It is possible to merge at most eight rows of synapses to achieve a fan-in of 1 k plastic synapses and 512 non-plastic ones per neuron, for a network of eight usable neurons.

The spikes generated by the neurons get encoded into AEs by the 1D arbiter. The LUT is used to append a destination chip and core address to each AE. There can be up to eight different copies of an AE with eight different destination addresses, to increase the fan-out of each neuron and allow it to target up to eight different chips and maximum 32 cores. The local R1 router then routes the AEs to the corresponding destinations. Once an AE reaches its destination core, the address gets broadcast to the whole core (which comprises 256 neurons). So, in principle it is possible to achieve a maximum fan-out of 8 k destinations.

### Features of the Dynap-SEL System

Thanks to the flexibility of the memory-optimized routing system adopted, the system is highly scalable (Moradi et al., 2015). Resources from different chips can be easily combined and merged. Plastic cores from up to 4 × 4 chips can be merged together to build a larger core. For example, by merging plastic cores from 16 chips, a plastic core with 128 × 1 k plastic synapses and 1 k neurons, or a plastic core with 1 k × 128 plastic synapses and 128 neurons can be configured. The implementation of asynchronous memory control allows the on-line re-configuration of the routing tables. This in turn allows the implementation of structural plasticity or evolutionary algorithms.

**Figure 17:**
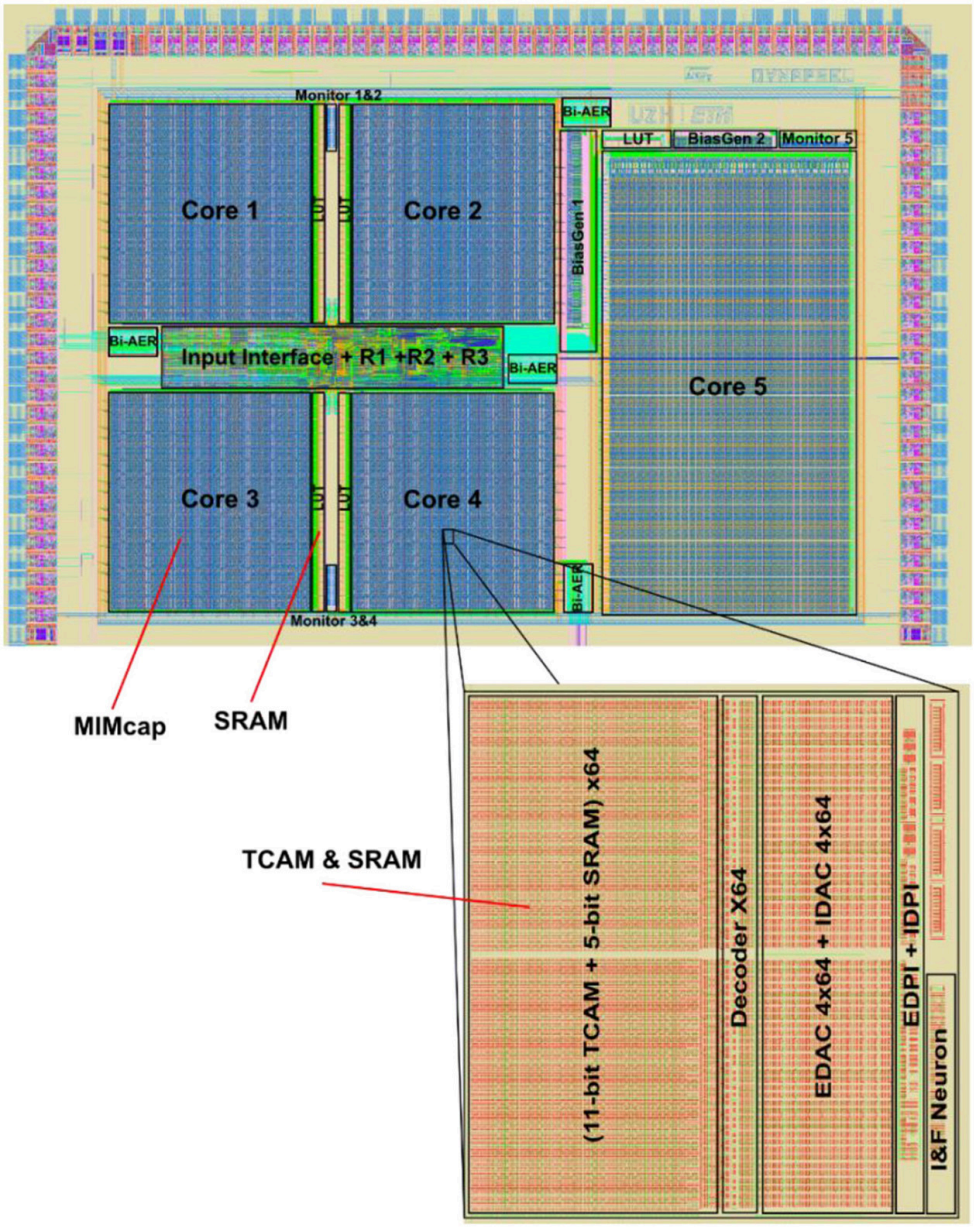
Dynap-SEL chip, fabricated using a 28 nm FDSOI process. It occupies an area of 7.28 mm^2^ and comprises four non-plastic cores and one plastic core. Each non-plastic core has 256 analog I&F neurons and 64k TCAM-based synapses arranged in 2D array (each synapse address is identified by an 11-bit TCAM, and each synapse type and weight areidentified by a 5-bit SRAM). Each plastic core has 64 analog I&F neurons, 8k digital plastic synapses, 4k digital non-plastic synapses, and 256 virtual synapses.

By using multiple physical circuits to carry out parallel computation, and by implementing many small and distributed memory structures embedded within the mixed-signal neural processing fabric, this architecture eliminates, by design, the von Neumann bottleneck problem at the source. Memory and computation are co-localized at all levels of the hierarchy (Figure 17). In the current design, most of the silicon real-estate is occupied by the digital SRAM and TCAM memory cells. The use of emerging memory technologies, such as ReRAM, will allow to dramatically reduce the size of the design, and to integrate on the same area larger numbers of synapses and neurons.

### Application

The chip can find applications in various domains such as bio-signal processing for auditory keyword recognition, body signal processing like ECG anomaly detection based on reservoir computing, vision processing applications for face detection and object tracking and obstacle avoidance with robotics systems.

## The 2DIFWTA Chip: a 2D Array of Integrate-And-Fire Neurons for Implementing Cooperative-Competitive Networks

The 2DIFWTA (2D Integrate-and-Fire Winner-Take-All) chip was developed in the lab of Elisabetta Chicca at the Cluster of Excellence in Cognitive Interaction Technology CITEC and Bielefeld University, Germany. In this chip, cooperative-competitive networks typically consist of a population of neurons with recurrent excitatory and inhibitory connections. The inhibitory connections mediate competition among neurons receiving different input stimuli, while recurrent excitation support cooperation among neurons with similar response properties (e.g., close receptive field or stimulus preference). The group of neurons with the highest response suppresses all other neurons and wins the competition. Cooperative-competitive networks perform complex non-linear operations as well as common linear operations. Representative examples of linear operations are analog gain (i.e., linear amplification of the feed-forward input, mediated by the recurrent excitation and/or the common mode input) and locus invariance (Koch and Segev, 1998). The non-linear operations include non-linear selection or soft winner-take-all (WTA) behavior (Amari and Arbib, 1982; Hahnloser et al., 2000; Dayan and Abbott, 2001), signal restoration (Douglas et al., 1994; Dayan and Abbott, 2001), and multi-stability (Amari and Arbib, 1982; Hahnloser et al., 2000; Dayan and Abbott, 2001). These operations are believed to be widespread in the nervous system, and the WTA architecture has been proposed as a computational primitive of the canonical microcircuit of the custom VLSI chip with a dedicated architecture for implementing a single 2D cooperative-competitive network neocortex.

A full-custom VLSI chip with a dedicated architecture for implementing a single 2D cooperative-competitive network or multiple 1D cooperative-competitive networks has been implemented. The 2DIFWTA chip was explicitly designed to provide a tool for the exploration of cooperative-competitive network dynamics and computational properties in both the mean rate and time domain. Recurrent connections are internally hard-wired and do not need to be routed through the AER bus. Similar architectures lacking these internal connections would easily experience an AER bus overload, resulting in prohibitive latencies, when configured to realize the same density of recurrent connections.

The neurons and synapses embedded in the chip are sub-threshold analog circuits for real-time emulation of the chosen models. The system is fully parallel, therefore large-scale versions can be designed without affecting the real-time operation of the system. Nevertheless, suitable modular structures must be identified to guarantee low traffic on the AER bus.

### Chip Description

**Figure 18:**
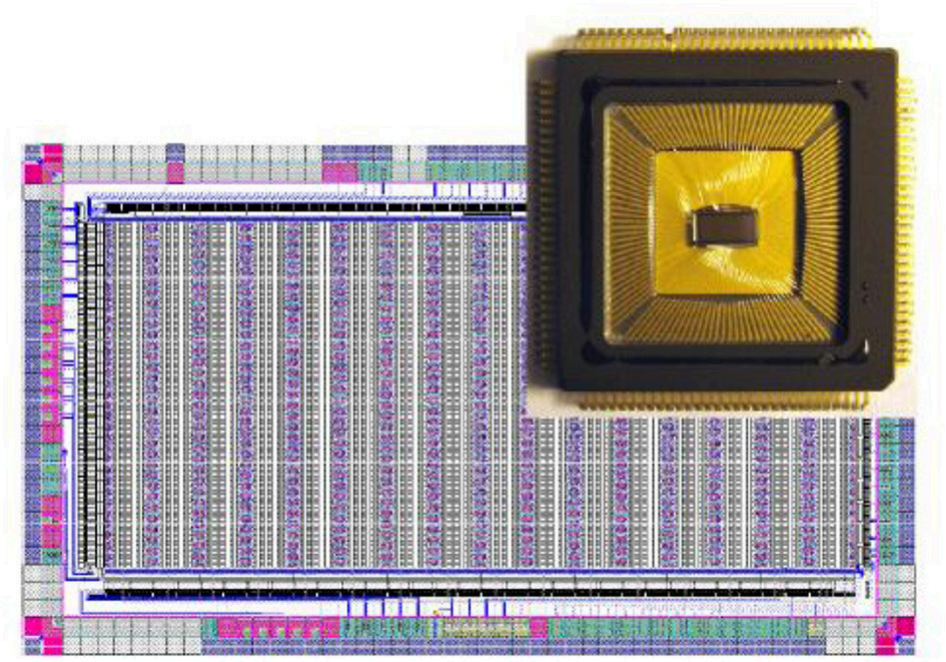
2DIFWTA chip layout and photograph. The 2DIFWTA chip was implemented using a standard 0.35-μm four-metal CMOS technology and covers an area of about 15 mm^2^.

**Figure 19:**
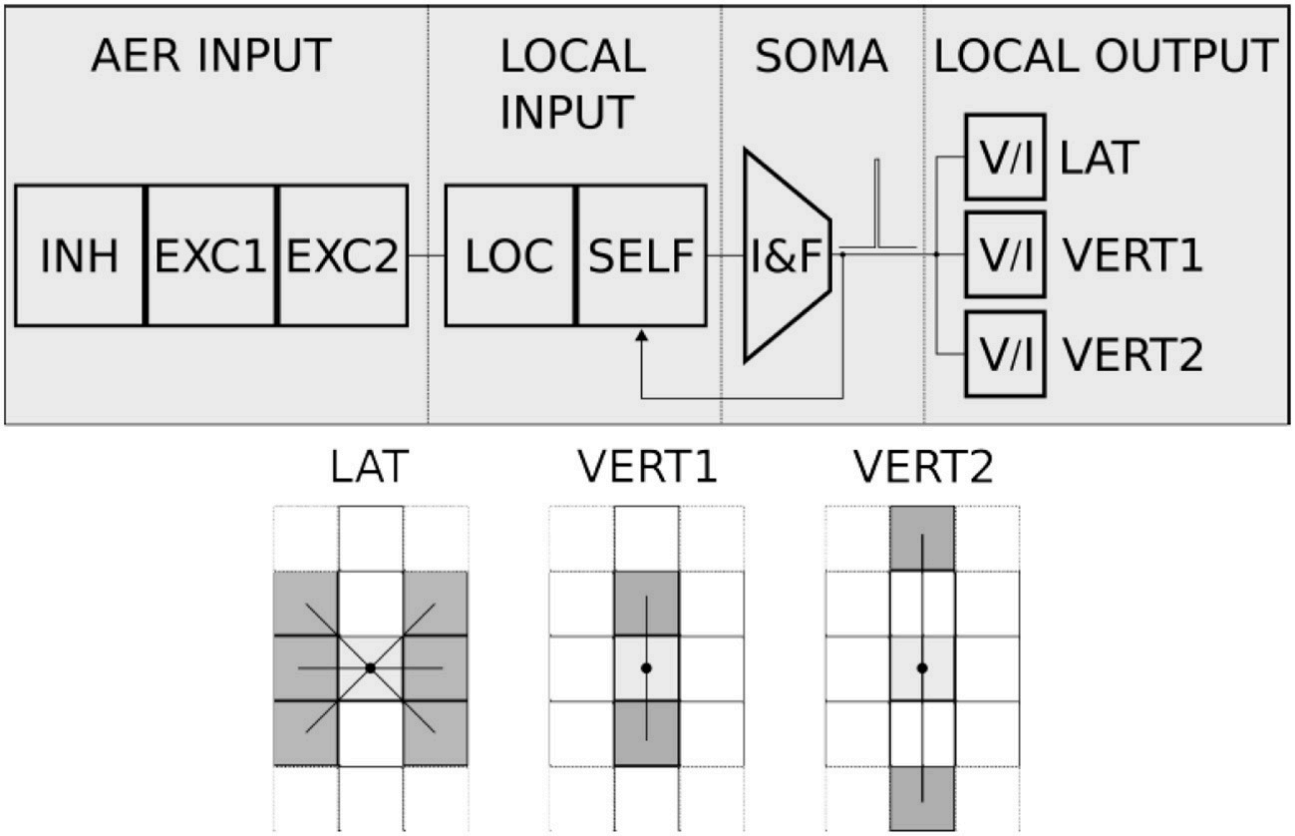
Neuron diagram (top) and local connectivity (bottom). Each neuron comprises the following blocks: AER input, local input, soma and local output. The AER input consists of three AER synaptic circuits: two excitatory and one inhibitory. The local input comprises a diff-pair circuit to integrate all the local excitatory input (see local connectivity diagram below) and an excitatory synapse to implement self-excitation. The soma is an I&F neuron circuit that integrates the sum of all currents generated by the AER input and local input blocks. The local output block generates pulse currents for the local input blocks of the neighbors' neurons. The pattern of recurrent local connectivity is represented in the bottom diagram. Local cooperation of the 2D network is implemented by activating the lateral (“LAT”) and vertical to first neighbors' (“VERT1”) local connections. Several 1D networks with first- and second-neighbor cooperation are implemented by activating the vertical to first neighbors' (“VERT1”) and vertical to second neighbors' (“VERT2”) recurrent excitatory connections.

The 2DIFWTA chip was implemented using a standard 0.35-μm four-metal CMOS technology (Figure 18). It comprises a two-dimensional array of 32 × 64 (2,048) I&F neurons. Each neuron (Figure 19) receives inputs from AER synapses (two excitatory and one inhibitory) and local excitatory synapses. The local connections implement recurrent cooperation for either a two-dimensional or 32 mono-dimensional WTA networks. Cooperation in 2D involves first-neighbor connections, while cooperation in 1D involves first- and second-neighbor connections. Competition has to be implemented through the AER communication protocol, and it is therefore flexible in terms of connectivity pattern.

### Neuron (I&F) Model

**Figure 20:**
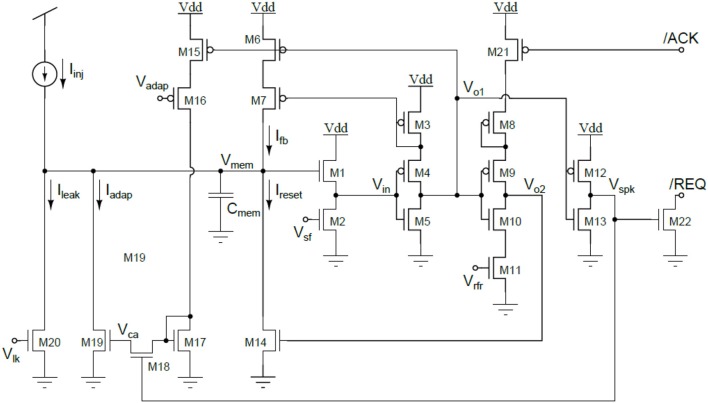
Circuit diagram of the I&F neuron. The neuron's membrane capacitor *C*_*mem*_ integrates the input current *I*_*inj*_ into the membrane voltage *V*_*mem*_. When*V*_*mem*_reaches the spiking threshold *V*_*spk*_ the occurrence of a spike is signaled by the output voltage *V*_*spk*_, which quickly switches for zero to the power supply rail. The reset current *I*_*reset*_ is activated by *V*_*o*2_ and discharges the membrane capacitor. The input current can be integrated again after the refractory period, which duration is set by the bias voltage *V*_*rfr*_ (low *V*_*rfr*_values lead to long refractory period durations). A source follower circuit (M1 and M2) is used to modulate the spiking threshold. A firing rate dependent current *I*_*adap*_ is subtracted to the input current to implement spike frequency adaptation (M15-19). The amplitude of this current increases with each output spike (the increase rate is set by the bias voltage *V*_*adap*_) and decreases exponentially with time. Power consumption is drastically reduced owing to a positive feedback module (M3, M6-7) that reduces the duration of the transition period in which the inverters switch polarity. The circuit's bias voltages (*V*_*lk*_, *V*_*adap*_, *V*_*alk*_, *V*_*sf*_, and *V*_*rf*_) are set to operate the transistors in the sub-threshold region and they determine the neuron's properties.

The circuit diagram of the I&F neuron implemented on the 2DIFWTA chip is shown in Figure 20. This circuit implements a leaky I&F model similar to the design proposed in Culurciello et al. (2001). Several biologically realistic features are also implemented: refractory period, spike-frequency adaptation, and threshold voltage modulation. All circuit parameters are tunable, thanks to external bias voltages. A detailed description of the circuit operation can be found in Indiveri et al. (2006).

### Synapses

The synaptic circuit implemented in this chip is the one proposed by Bartolozzi and Indiveri (2007) and referred to as the DPI synapse. The primary motivation for choosing this synaptic circuit for the 2DIFWTA chip relates to its linear filtering properties. The two-dimensional structure of this chip implies strong limitation on the space available for synaptic circuits provided the goal of integrating a large number of neurons. The linearity of the synaptic circuit allows multiplexing of different spiking sources, so that a single synaptic circuit can virtually act as multiple synapses. This property is used in the 2DIFWTA chip both for the input AER connections and the local connections, whenever a single time constant can be accepted.

### Full Neuron and Local Connectivity

The full neuron comprises several blocks, as depicted in Figure 19. The two AER excitatory synapses labeled “EXC1” and “EXC2” have independent bias settings and are intended for providing external stimuli or implementing arbitrary recurrent connections. The AER inhibitory synapse (“INH”) can be used to transmit global inhibition signals in a cooperative-competitive network configuration, but it is also suitable for providing any external and recurrent inhibitory input. The local input block comprises an “LOC” circuit for summing up all contributions from local cooperative (e.g., excitatory) connections and integrating them through a DPI synapse (see section Synapses). The “SELF” block implements self-excitation. The “I&F” circuit is described in section Neuron (I&F) model. The local output generates contributions for neighbor neurons (to be integrated in the respective “LOC” block). The connectivity scheme for the recurrent excitation (as depicted in the bottom part of Figure 19) has been designed to provide two kinds of cooperative-competitive networks. The “LAT” and “VERT1” connections can be activated simultaneously to implement local cooperation in a 2D network. Otherwise, the “VERT1” and “VERT2” connections can be activated simultaneously for implementing 32 1D networks of 64 neurons with first- and second-neighbor excitatory recurrent connections. All synaptic circuits included in the “AER INPUT,” “LOCAL INPUT,” and “LOCAL OUTPUT” blocks can be independently activated or de-activated, therefore providing full flexibility for the effective connectivity matrix of the 2,048 neurons.

### Application

Despite the very specific motivation of designing the 2DIFWTA chip for implementing cooperative-competitive networks, its architecture is such that the chip can be treated as a general-purpose, large-scale pool of neurons. Therefore, it has been possible to use this chip to address a broad range of research questions, as demonstrated by the number of publications making use of this hardware.

The 2DIFWTA chip was used to evaluate the role of global inhibition in a hardware model of olfactory processing in insects (Beyeler et al., 2010). Function approximation (Corneil et al., 2012a) and inference (Corneil et al., 2012b) were implemented on neuromorphic hardware by making use of multiple 1D cooperative-competitive networks available on the 2DIFWTA chip. Both first- (“VERT1”) and second-neighbor (“VERT2”) recurrent excitatory connections were activated for these experiments.

In Sheik et al. (2012a), the chip was used as a large pool of neurons (with no recurrent connections) configured to maximize mismatch in the generation of the action potential in response to a single input pulse, therefore producing a distribution of axonal delays in the range of milliseconds. This result was instrumental in the later implementation of a neuromorphic system for unsupervised learning of simple auditory features (Sheik et al., 2012b).

Probably the most advanced application of this chip was presented in Neftci et al. (2013), where a real-time neuromorphic agent able to perform a context-dependent visual task was demonstrated. The paper presented a systematic method for configuring reliable Finite State Machine (FSM) computation on spiking neural arrays implemented on neuromorphic hardware.

The 2DIFTWA was later used to explore models of auditory perception in crickets (Rost et al., 2013) and to study latency code processing in weakly electric fish (Engelmann et al., 2016).

## PARCA: Parallel Architecture With Resistive Crosspoint Array

**Figure 21:**
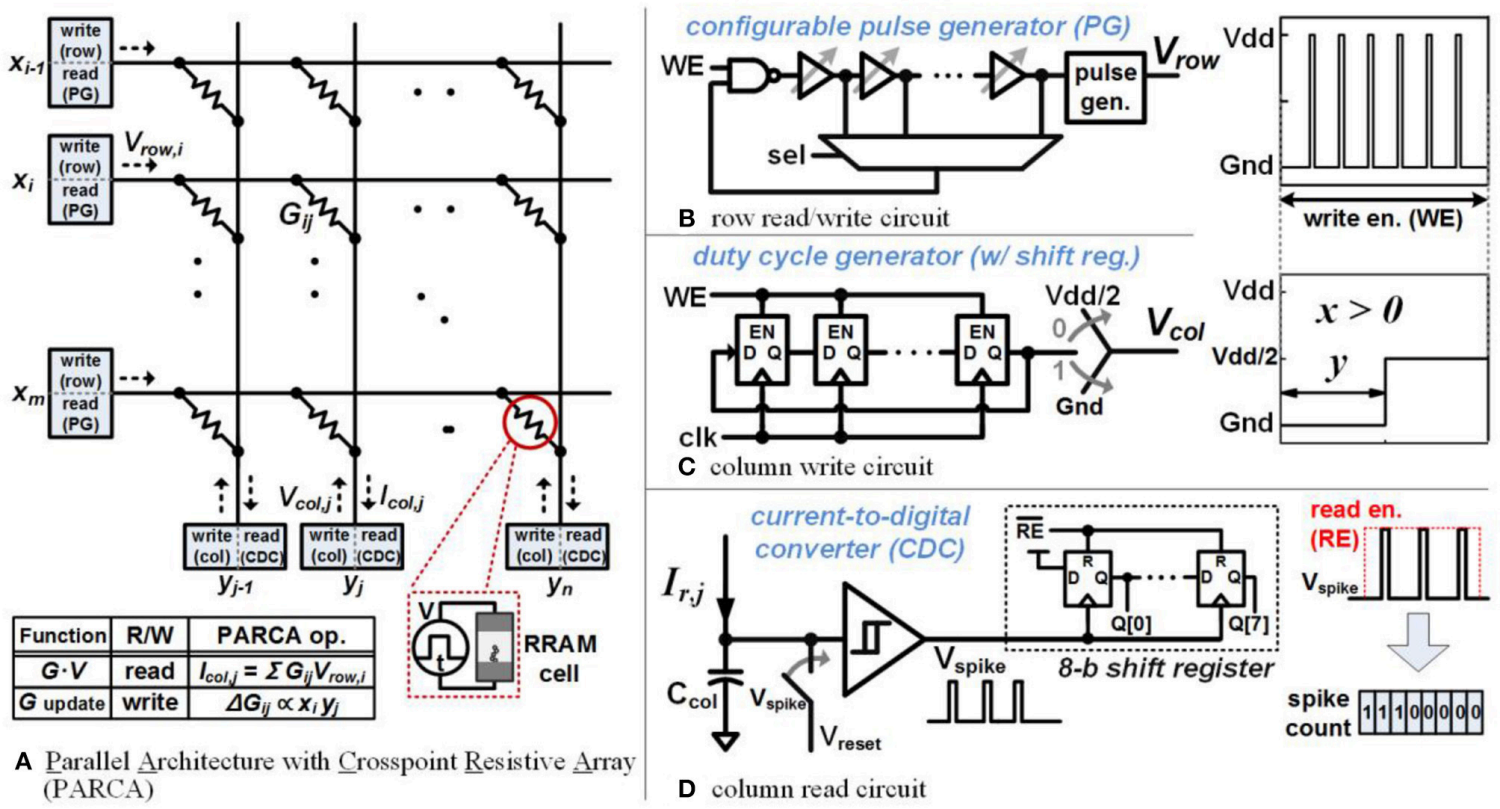
Parallel architecture with resistive crosspoint array (PARCA). **(A)** Parallel architecture of resistive crosspoint array (PARCA) is shown. **(B)** Row read/write circuit modulates the number of pulses over time. **(c)** Column write circuit generates the duty cycle window where write is effective. **(D)** Column read circuit converts analog output current (weighted sum) into digital values. Adapted from (Seo et al., 2015).

The parallel architecture with resistive crosspoint array (PARCA) (Seo et al., 2015), developed in the lab of Yu Cao at the Arizona State University, employs read-and-write circuits on the periphery (Figure 21A). Integration of resistive synaptic devices into crossbar array architecture can efficiently implement the weighted sum or the matrix-vector multiplication (read mode) as well as the update of synapse weight (write mode) in a parallel manner. PARCA replaces the sequential row-by-row operations in the CMOS ASIC SRAM with a massively parallel read-and-write operation using resistive-RAM (RRAM) cells for image/speech recognition applications. Using resistive devices for the synapses/weights in neural networks could substantially reduce the memory area and benefit additional speed-up due to further parallelization. Employing special read-and-write circuits on the periphery of the array, matrix-vector multiplications can be performed fully in parallel on an RRAM array by a combination of current-summing and time/voltage modulation on the periphery.

### PARCA Operations and Test Chip Implementation

To compute the weighted sum, the RRAM array is operated in the read mode. Given an input vector *x*, a small read voltage *V*_*row, i*_ is applied simultaneously for every non-zero binary bit of *x* (Figure 21B). *V*_*row, i*_ is multiplied with the conductance *G*_*ij*_ at each crosspoint, and the weighted sum results in the output current at each column end. As shown in Figure 21D, the column read circuit integrates this analog current and converts to spikes or digital output values (i.e., current-to-digital converter) with a non-linear activation function (e.g., thresholding), which mimics the integrate-and-fire functionality of spiking neurons in our brain. Note that the proposed architecture performs analog computing only in the core of the crossbar array, and the communication between arrays is still in a digital fashion. Compared to conventional memories that require row-by-row read operation; this approach reads the entire RRAM array in parallel, thereby accelerating the weighted sum.

To update the resistive synapse weights, the RRAM array is operated in the write mode, with local programming voltage generated at local row and column peripheries (Figures 21C,D). This approach can implement the stochastic gradient descent (SGD) or spike-based learning algorithm where the intended synapse weight change is mapped to the conductance value change of RRAM devices. The column write circuit (Figure 21C) generates a programming pulse with a duty cycle proportional to column neuron value. The row write circuit (Figure 21B) generates a number of pulses proportional to the row neuron value, where the pulse width is fixed and the pulses are evenly distributed across a constant write period to minimize the quantization error. During the write period, the conductance of RRAM cells is changed by the aggregate overlap time of the column write window (*y*_*j*_) and pulses on the row side (*x*_*i*_), which effectively represents *x*_*i*_*y*_*j*_.

**Figure 22:**
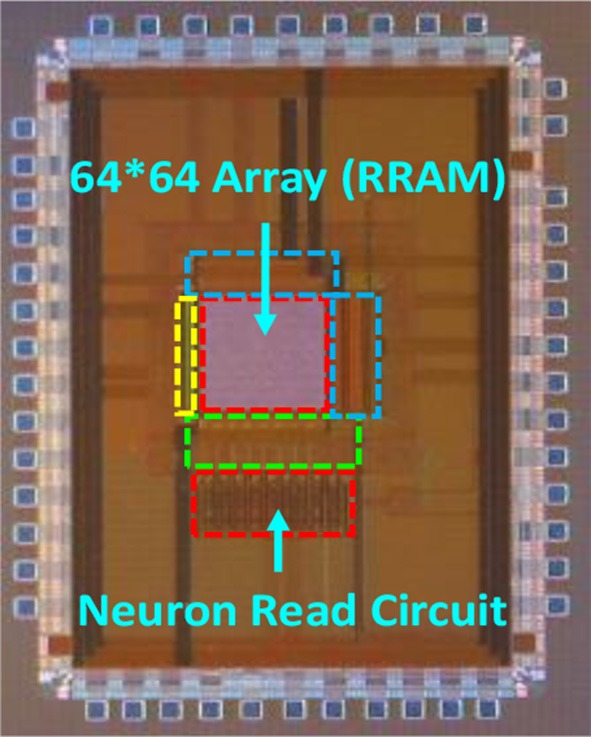
Crossbar macro die.

So far, there have been a few experimental implementations of simple algorithms on small-scale crossbars: 40 × 40 Ag:a-Si crossbar (loading weights without learning) (Kim et al., 2012), 12 × 12 TiO_x_/Al_2_O_3_ crossbar (simple perceptron algorithm with software neurons) (Prezioso et al., 2015), IBM's 500 × 661 PCM 1T1R array (multi-layer perceptron with software neurons) (Burr et al., 2014), and IBM's 256 × 256 PCM 1T1R array (with on-chip integrate-and-fire neurons) (Kim et al., 2015). Recently, a 64 × 64 neurosynaptic core with RRAM 1T1R synaptic array and CMOS neuron circuits at the periphery was designed and fabricated, as shown in Figure 22. The RRAM is monolithically integrated between M4 and M5 in a 130 nm CMOS process. In this design, the RRAM is a conventional binary device, suitable for binary neural networks. The CMOS neurons at column peripheries have been time-multiplexed to minimize neuron area and sequentially read out different columns.

### Mitigating Non-ideal Effects of Resistive Synaptic Devices

**Figure 23:**
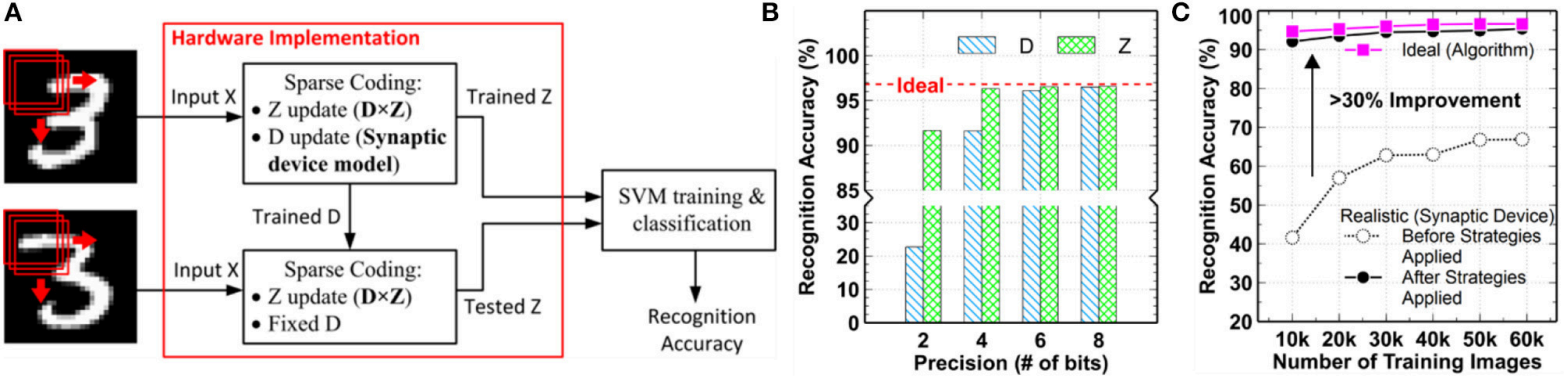
Device-algorithm co-simulation flow. **(A)** Flow of sparse coding with non-ideal effects modeled in the algorithm. **(B)** Recognition accuracy as a function of precision of bits of the neuron vector (Z) and weight matrix (D). **(C)** Recognition accuracy with realistic device behaviors and the improvement by the proposed mitigation strategies. Adapted from (Chen et al., 2015a,b).

Today's resistive synaptic devices with multilevel states could emulate the analog weights in the neural network and the crossbar array could efficiently implement the weighted sum and weight update operations. However, non-ideal device effects exist when mapping the weights in the algorithms into the conductance of the devices, including: (1) precision (or number of levels) in the synaptic devices is limited as opposed to the 64-bit floating-point in software; (2) weight update (conductance vs. # pulse) in today's devices is nonlinear and asymmetric [see representative examples of PCMO (Park et al., 2013), Ag:a-Si (Jo et al., 2010), and TiO_2_/TaO_x_ (Gao et al., 2015) devices in literature]; (3) weight on/off ratio is finite as opposed to the infinity in software, as the off-state conductance is not perfectly zero in realistic devices; (4) device variation, including the *spatial* variation from device to device and the *temporal* variation from pulse to pulse, is remarkable; (5) at array-level, the IR drop along interconnect resistance distorts the weighted sum. To evaluate the impact of non-ideal effects of synaptic devices and array parasitics on the learning accuracy at the system-level, it is necessary to formulate a methodology to abstract the device's behavioral model and incorporate it into key operation steps in the algorithm. Pioneering works (Chen et al., 2015a,b) have been performed to study the non-ideal device effects using sparse coding algorithm as a case study. Sparse coding is an unsupervised learning algorithm for efficient feature extraction and it is bio-plausible: neurons in the visual cortex can form a sparse representation of natural scenes (Olshausen and Field, 1996). The device-algorithm co-simulation flow is shown in Figure 23A. The MNIST handwritten digits dataset (LeCun et al., 1998) was used for benchmarking. Figure 23B shows the learning accuracy with different precisions by truncation of the bits of the neuron vector (Z) and weight matrix (D) in the algorithm. At least 6-bit D and 4-bit Z are needed for high learning accuracy. This translates to 64 levels of conductance in synaptic devices, which is available in today's devices. However, with a naïve implementation of today's realistic synaptic devices, it only achieves a poor recognition accuracy (~65%) of the MNIST handwritten digits compared to that with ideal sparse coding algorithm (~97%), as shown Figure 23C. The impact of the non-ideal effects is evaluated one by one, and it is summarized in Table 4.

**Table 4:** Summary of non-ideal effects and mitigation strategies (Adapted from Chen et al., 2015a,b).

**Non-ideal effects**	**Impact on learning accuracy**	**Mitigation strategies**
Limited weight precision	Significant when < 32 levels	Need >64 levels by device engineering
Non-linear weight update	5% drop is non-linearity is large (when ~1/3 number of pulses is enough to increase the weight from 0 to 90% full)	Smart programming scheme
Limited on/off ratio	~20% drop when on/off ratio < 15	Pacifier column and differential read-out
Device spatial variation	Not significant even when variation is 30%	Multiple cells as one-bit to average out variation by redundancy
Device temporal variation	~20% drop when variation is 30%
IR drop on the array wire	>7% drop is wire width < 50 nm for 900 ^*^ 300 array	Relax the wire width

To mitigate these non-ideal device effects, strategies at circuit- and architecture-level were proposed (Chen et al., 2015a,b), and are also summarized in Table 4. These include a pacifier column to eliminate the off-state current, smart programming schemes to improve the linearity of weight update, the use of multiple cells as one-weight bit to statistically average out device variations, and relaxing the wire width for reducing the IR drop. With the proposed strategies applied, the recognition accuracy can increase back to ~95%. These mitigation strategies are associated with penalty of area, power, and latency, etc.

### Application

The system targets image and speech processing applications. For example, image/digit recognition (Seo et al., 2015; Sun et al., 2018) and language modeling have been demonstrated by employing a number of multiple arrays of this chip, where the inter-array communication/computation are performed in digital signals.

## Transistor-Channel-Based Programmable and Configurable Neural Systems

This section discusses the neural system design and potential of transistor-channel modeling. Modeling of biological dynamics in physical implementation results in practical applications, consistent with Mead's original vision (e.g., Mead, 1989a). One does not require choosing modeling neurobiology or building practical applications. Neurobiology seems optimized for energy-efficient computation, something engineers explicitly want for embedded applications. Long-term memory elements enable computation as well as robustness from mismatch. These approaches can become the basis for building models of the human cortex as well as having impact for commercial applications (Hasler and Marr, 2013).

### Transistor-Channel Models of Neural Systems

**Figure 24:**
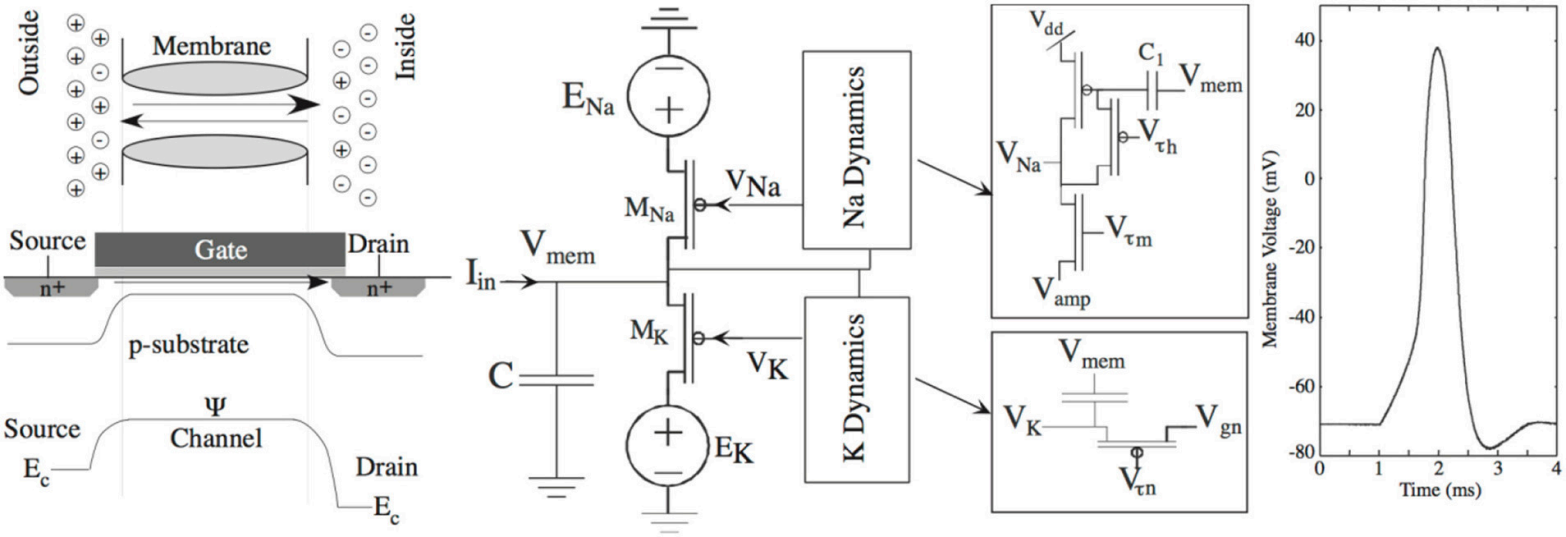
An overview of MOSFET channel modeling of biological channels. This approach is possible given the similar (although not identical) physics between MOSFET and biological channels, both modulated by a gating potential. The physical structure of a biological channel consists of an insulating phosphor-lipid bilayer and a protein that stretches across the barrier. The protein is the channel in this case. The physical structure of a MOSFET consists of polysilicon, silicon dioxide, and doped n-type silicon. A channel is formed between the source and the drain. The band diagram of silicon has a similar shape to the classical model of membrane permeability. This approach yields an updated model for modeling biological channels that also empowers dense MOSFET implementation of these approaches. The primary design constraint is modeling the gating function with other transistor devices; such an approach is shown to model the classic Hodgkin–Huxley squid axon data, resulting in a close model to the event, as well as voltage clamp experiments.

Transistor-channel models of neural systems were developed in the lab of Jennifer Hasler at the Georgia Institute of Technology. The base components are based on transistor-channel models of biological channel populations (Farquhar and Hasler, 2005) (Figure 24). The physical principles governing ion flow in biological neurons share similarities to electron flow through MOSFET channels, and exploiting these similarities results in dense circuits that effectively model biological soma behavior. The energy band diagram (source to drain) looking through the channel of the MOSFET is similar to the energy band diagram (inside to outside) looking through a biological channel. Because the similarities between biological and silicon channels are utilized, the voltage difference between the channel resting potentials on the silicon implementation is similar to that in the biological power supplies. The resulting spiking action potential circuit requires six transistors (Figure 24), which is the same number of transistors and just a few more capacitors (transistor-size capacitors) than in the basic integrate-and-fire neuron approach (Mead, 1989a).

### Long-Term Analog Memories: Dense Silicon Synapse Models

**Figure 25:**
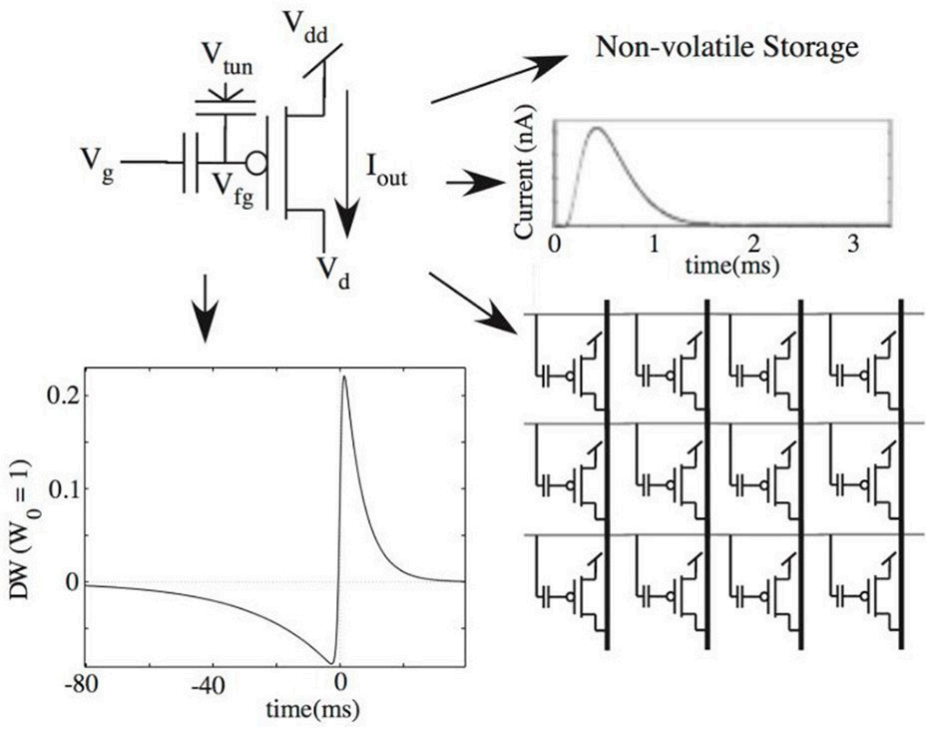
A single transistor synapse device is presented;the architecture uses non-volatile storage, generates biological post-synaptic potential (PSP) outputs, can easily be arrayed in a mesh architecture, and demonstrates biological synapse learning rules such as long-term potentiation (LTP), long-term depression (LTD), and spike time dependent plasticity (STDP).

Long-term memory enables computation, including physical computation, which encompasses analog and neuromorphic computation. A FG device is employed that can be used to store a weight in a non-volatile manner, compute a biological excitatory post-synaptic potential (EPSP), and demonstrate biological learning rules (Figure 25) (Hasler et al., 1994; Gordon et al., 2004; Ramakrishnan et al., 2011).

Synapses represent the connection between axon signals and the resulting dendrite of a particular neuron. The connection starts as an electrical event arriving into the pre-synaptic cell, releasing chemicals that reach and modulate the channels at the post-synaptic cell, resulting in a response in the dendritic structure. Figure 25 shows single transistor learning synapse using a triangle waveform modeling the pre-synaptic computation, a MOSFET transistor modeling the post-synaptic channel behavior, and anFG to model the strength of the resulting connection. A MOSFET transistor in sub-threshold has an exponential relationship between gate voltage and channel current, therefore achieving the resulting gate voltage to get the desired synapse current, which has the shape of a triangle waveform. These learning synapses have storage capabilities to enable them to retain 100s of quantization levels (7–10 bits), limited by electron resolution, even for scaled down FG devices (i.e., 10 nm process).

Biological synapses adapt to their environment of event inputs and outputs, where typical programming rules include long-term potentiation (LTP), long-term depression (LTD), and spike-time-dependent plasticity (STDP). A single FG device has enabled both the long-term storage and PSP generation, but also has allowed a family of LTP-, LTD-, and STDP-type learning approaches through the same device (Gordon et al., 2004). The weight increases when the post-synaptic spikes follow the pre-synaptic spikes and decreases when the order is reversed. The learning circuitry is again placed at the edges of the array at the end of the rows, included in the soma blocks, therefore not limiting the area of the synaptic matrix/interconnection fabric.

### Neuromorphic IC of Somas and Learning Synapses

**Figure 26:**
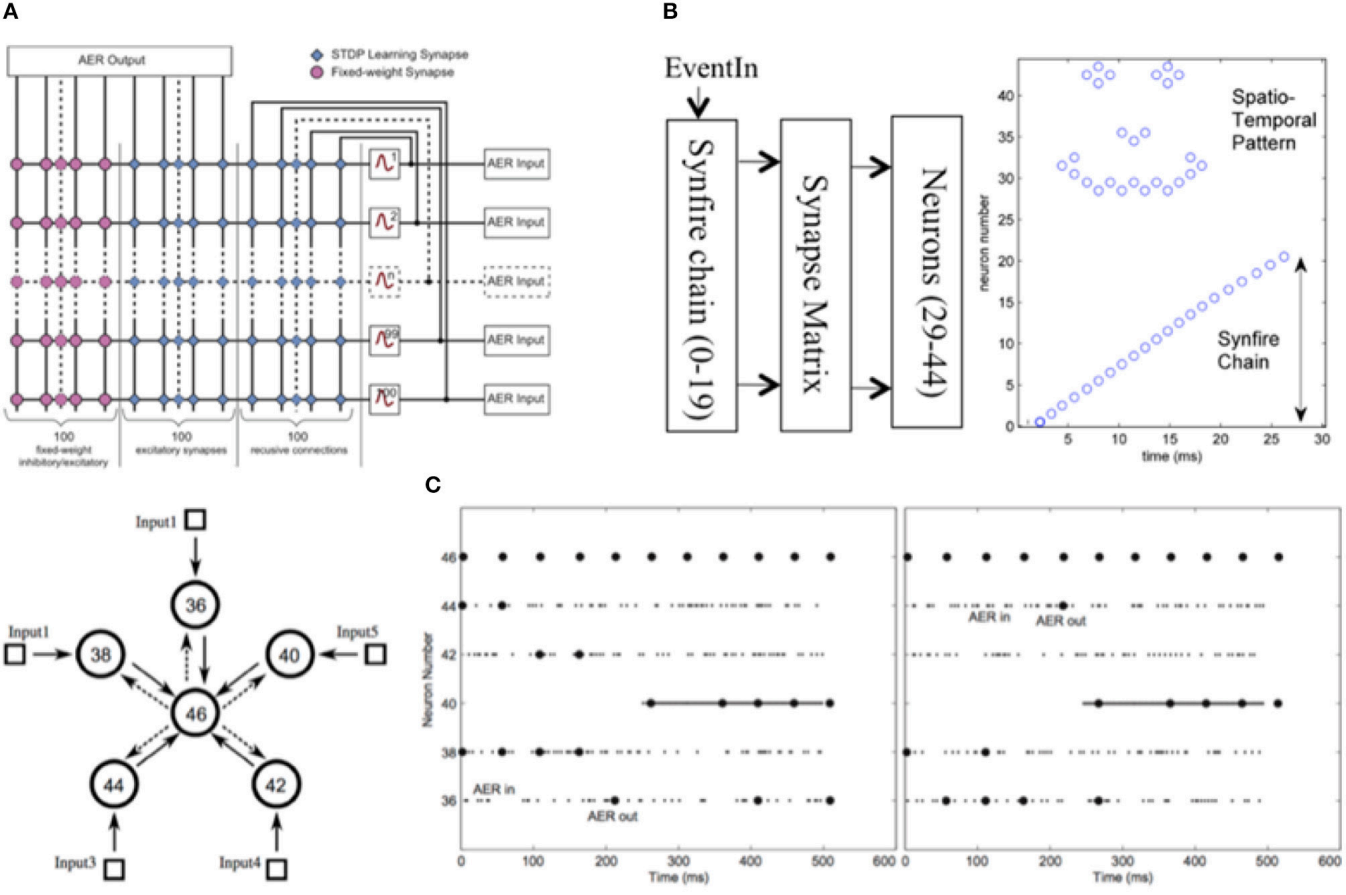
Neuron IC built from transistor-channel-modeled components. **(A)** The IC uses a mesh-type structure of synapses, an array of configurable elements to compile the desired soma dynamics, and AER blocks that output action potentials into the synapse fabric as well as AER blocks that that input action potentials from soma blocks, 10,000 STDP excitatory learning enabled synapses connected in recurrent connections, 10,000 STDP excitatory learning enabled synapses connected from AER, and 10,000 programmable (fixed-weight) excitatory or inhibitory synapses connected from AER. **(B)** Measured synfire neuron chain activating other programmed neurons. **(C)** Diagram and experimental measurements of a spiking Winner-Take-All (WTA) network drawn as a ring topology. The network is a combination of input neurons on the outside part of the ring, and an interneuron, which provides the negative feedback through inhibitory neurons, for the WTA computation. This work labels the particular neurons used for this experiment, and all synapses used are programmable synapses, whether excitatory or inhibitory.

Figure 26 shows an IC implementation of biological model circuits for synapses, soma (channel model), and input and output spikes efficiently into the system (Brink et al., 2013). A mesh architecture (or crossbar, as originally described in Hasler et al., 1994) enables the highest synapse array density interconnecting configurable, channel-neuron model components. The soma has the capability of multiple channels, infrastructure for communicating action potential events to other structures, as well as circuits to build local WTA feedback between the soma membrane voltages. This configurable array is a specialized large-scale Field Programmable Analog Array (FPAA) (e.g., George et al., 2016). The array of synapses compute through a memory that is similar to an array of EEPROM devices; any off-synapse memory-based scheme will only be more complex and expensive system design. The chip uses AER, with the digital computation synthesized from Verilog using standard digital cells. The synapses were enabled for STDP learning operation and initial learning experiments performed (Nease et al., 2013).

Figure 26 shows an example of a synfire chain of neurons. These neurons were programmed with some mismatch compensation, although the channel models were not programmed using mismatch compensation. Additional neurons can be used for additional computations, including showing an artificial pattern from spikes.

Figure 26C shows a WTA topology composed of multiple (5) excitatory neurons that all synapse (excitatory synapses) onto a single neuron, which provides inhibitory feedback connection to all of the original neuron elements. The strength of the excitatory connection between each excitatory neuron and the center inhibitory neuron is programmed identical for all excitatory neurons. The strength of the inhibitory connection between the center inhibitory neuron and each excitatory neuron is programmed to identical values, although the strength and time duration of the inhibition time was stronger than the excitatory inputs.

### Application

**Figure 27:**
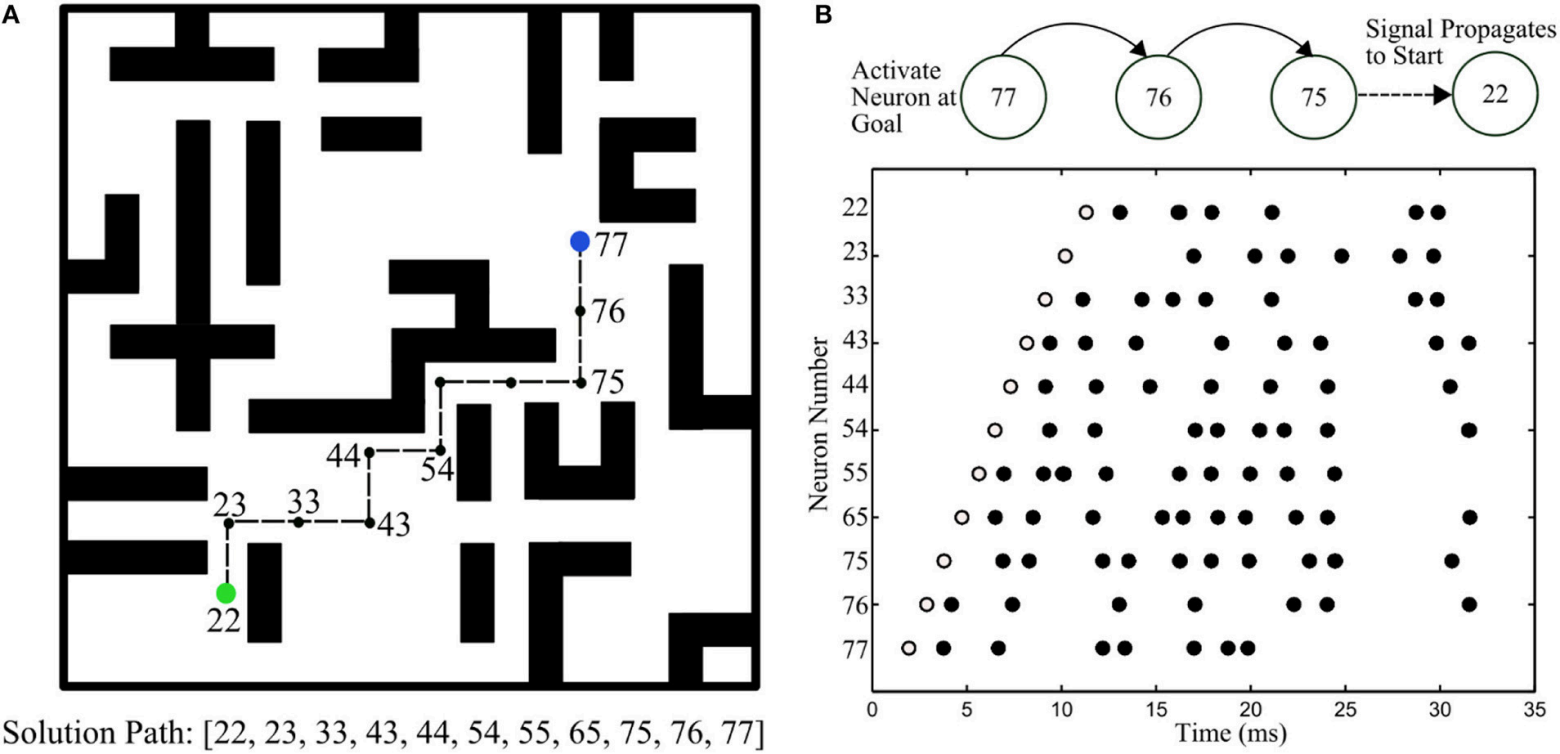
Optimal path planning originally demonstrated on the IC in Figure 26. **(A)** Grid environment example that the neuron IC solved. The device is located at Node 22, and the goal is at Node 77. A wavefront in initiated at the goal (Node 77) and propagated throughout the neuron grid. **(B)** Raster plot of the solution nodes. Neuron 77 causes neuron 76 to fire, which causes neuron 75 to fire, etc.

This IC was used to demonstrate optimal path planning based on realistic neurons (Koziol et al., 2014). Others (e.g., Krichmar, 2016) have built on these approaches. Figure 27 shows how a maze environment is modeled using the neuron IC. A wave front was initiated at the goal (Node 77) and propagated throughout the neuron grid. Figure 27 shows a raster plot of the solution nodes. Neuron 77 causes neuron 76 to fire, which causes neuron 75 to fire, and so forth. Theoretical *and* experimental results show 100% correct and optimal performance for a large number of randomized maze environment scenarios. The neuron structure allows one to develop sophisticated graphs with varied edge weights between nodes of the grid. Neuron IC's planner analytical time and space complexity metrics were verified against experimental data.

### Reconfigurable Neuromorphic ICs of Dendrites, Learning Synapses, and Somas

**Figure 28:**
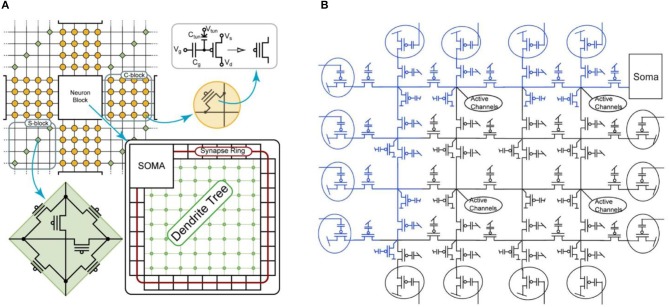
Neuron IC embedded in a typical FPAA/FPGA routing fabric. **(A)** The Neuron I/O interface with the routing at the C-Blocks through programmable floating-gate switches. The tracks are segmented for allowing faster event transmission and maximizing utilization. The tracks are routed at the S-Blocks, where each node consists of six switches. The neuron cell has synaptic inputs, programmable dendrites with active channels that aggregate inputs into the soma block. **(B)** Depiction of the neuron cell structure, with arbitrary dendritic structure capability. Dendrites are also interspersed with active channels to model the nonlinear behavior observed in biology. An 8-tap dendritic line programmed on the neuron is shown in blue color.

Figure 28 shows a further neuromorphic IC design now including dendritic computation, as well as FPAA re-configurability, into the resulting architecture. In many modeling and implementation approaches, the dendrite is approximated to be a wire, greatly simplifying the resulting network and enabling a system that is tractable by a range of computational principles. Using channel model approaches, one can successfully build dense dendritic compartments and configurable structures (Farquhar et al., 2004) that compare well to classical models of dendrites (Nease et al., 2012).

Figure 28 shows the architecture from a large-scale IC enabling dendritic modeling in its computation. Adding dendritic elements changes the neural architecture away from a neat crossbar device alignment. Utilizing concepts from FPAA devices (e.g., George et al., 2016), a single neuron is implemented as a single component in the routing. Events can be routed to and from the resulting block as typical in FPAA devices. This approach allows for sparse as well as densely connected neuron infrastructures. The dendritic tree has active and passive channels as well as a reconfigurable soma described in the last subsection. The architecture allows high synapse density in a working neural array; learning algorithms can be localized to a given block. Signal lines can be routed to AE senders and receivers (one-dimensional) to interface with off-chip devices; most (discussed further in Hasler and Marr, 2013) neurons have only local connections.

**Figure 29:**
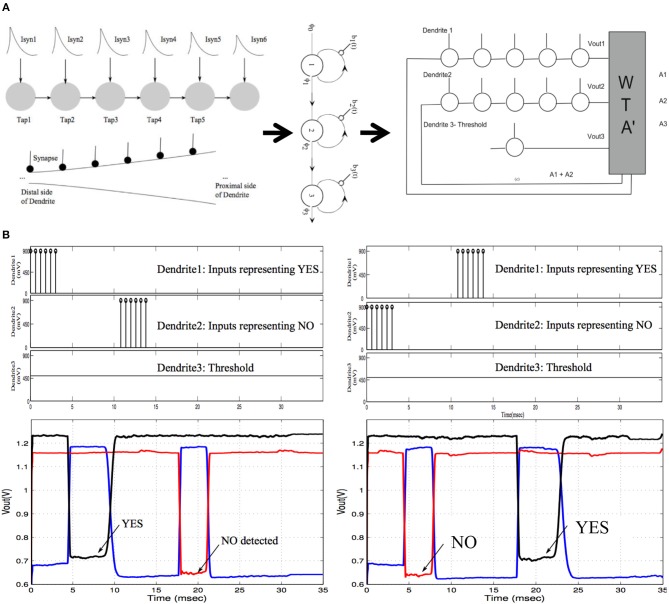
Dendritic computation using the framework in Figure 28. **(A)** Dendritic trees with increasing diameters have similar function to Hidden-Markov Model (HMM) trees used for acoustic classification. A YES-NO HMM classifier was built using dendritic lines to model linear HMM trees with a WTA terminating block. **(B)** Experimental demonstration of temporal classification using dendrite-enabled neurons to perform temporal classification (word spotting) similar to HMM classifier techniques. In the first case, YES is detected and then NO is detected, whereas in the second case, NO is detected and then YES is detected.

The resulting computation from dendritic elements is often debated, and in most computational models is ignored because of the huge increase in computational complexity. The computational efficiency of dendritic computation (Figure 29) has been demonstrated to be 1000s of times more efficient than that of most analog signal processing algorithms (George et al., 2013; Hasler and Marr, 2013). Dendritic structures can perform word-spotting, roughly approximating Hidden-Markov Model (HMM) classifier structures (George et al., 2013). This computation is critical in several engineering applications and typically is seen as too expensive to directly use. With as energy constrained as the cortex would be, as well as the need in embedded electronics for energy-efficient computation, these opportunities cannot be ignored.

### Potential Directions of Channel Model Computation

Carver Mead hypothesized that physical (e.g., analog) computation would be at least 1,000 × lower energy compared to digital computation based on transistor counting arguments, an argument experimentally shown multiple times (e.g., George et al., 2016). The potential opportunity has started serious discussions of the capability of physical computing (e.g., Mead, 1990) building the computing framework, which up to now, has only been developed for digital computing.

### Application

**Figure 30:**
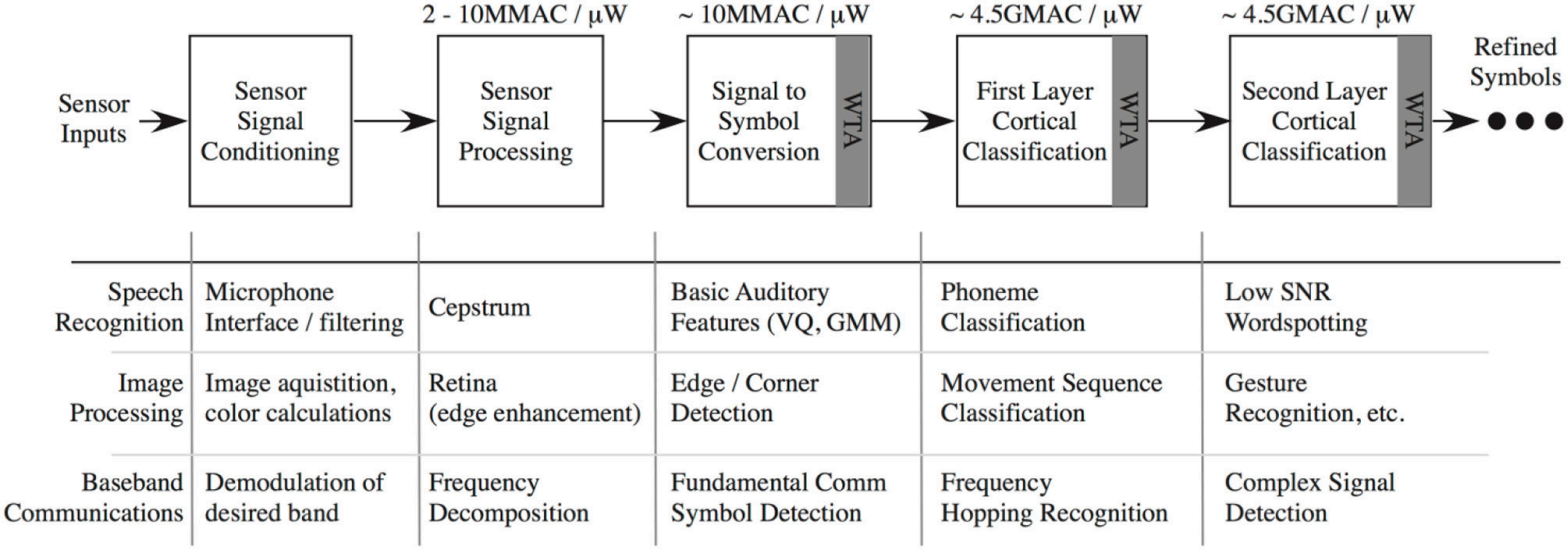
Typical signal processing chain using configurable analog approaches and neural-based classifiers.

The opportunities for neuromorphic computing to impact applications open up another level of energy-efficient computing. Figure 30 shows the possible opportunities in computing, enabling both analog as well as neuromorphic computing. Computation, classification, and learning systems that do not show sensor-to-output result capability give a myopic view of their results. These approaches enable opportunities toward building cortical structures (Hasler and Marr, 2013; Hasler, 2016) as well as enabling many new waves of computing opportunities.

## Other State-of-the-Art Neural Emulators

In this section, we briefly describe some of the well-known neural emulators. These emulators are already discussed in detail in Furber (2016), Benjamin et al. (2014), Merolla et al. (2011), Furber et al. (2014), and Davies et al. (2018) but we describe the main features of these emulators to provide a complete picture with respect to this review paper.

### Neurogrid

Neurogrid is a mixed-mode multichip system primarily used for large-scale neural simulations and visualization (Benjamin et al., 2014). The neuron circuits used in Neurogrid are closely correlated to the physical characteristics of neurons in the brain. It models the soma, dendritic trees, synapses, and axonal arbors. It consists of 16 neurocores/chips each with 65 k neurons (totalling 1 M neurons) implemented in sub-threshold analog circuits. A single neurocore is fabricated on an 11.9 × 13.9 mm die. A board of 16 neurocores is of size 6.5′′ × 7.5′′ and the complete board consumes roughly 3 W of power (a single neurocore consumes ~150 mW).

### TrueNorth

IBMs TrueNorth neuromorphic chip consists of 1 million digital neurons capable of various spiking behaviors (Merolla et al., 2011). Each die holds 4,096 cores, each core holding 256 digital neurons and 256 synapses per neuron. A single die consumes 72 mW of power. A board (NS16e) comprised of 16 TrueNorth chips has been developed; it consumes 1 W of power at 1 KHz speed, making it ideal for energy-efficient applications. Although digital in its implementation, low power consumption is due to fabrication in an aggressive, state-of-the-art 28 nm technology process.

### SpiNNaker

SpiNNaker (Furber et al., 2014), another digital neuromorphic neural array, was designed for scalability and energy-efficiency by incorporating brain-inspired communication methods. It can be used for simulating large neural networks and performing event-based processing for other applications. Each node is comprised of 18 ARM968 processor cores, each with 32 Kbytes of local instruction memory, 64 Kbytes of local data memory, packet router, and supporting circuitry. A single node consists of 16,000 digital neurons consuming 1 W of power per node. There exist two SpiNNaker circuit boards, the smaller being a 4-node (64,000 neurons) board and the larger a 48-node board (768,000 neurons). The 48-node board consumes 80 W of power.

### Loihi

The Loihi (Davies et al., 2018) is a neuromorphic chip introduced by Intel Labs. It simulates 130 K neurons and 130 M synapses in real time. The chip consists of 128 neuromorphic cores that are capable of on-chip training and inference. The hierarchical mesh protocol is implemented to support communication between the neuromorphic cores. Loihi is said to be the first fully integrated spiking neural network chip that supports sparse network compression, core-to-core multicast, variable synaptic format, and population-based hierarchical connectivity. Loihi contains epoc-based synaptic modification architecture in addition to pairwise STDP and Triplet STDP. Loihi includes computation blocks such as stochastic noise, which might be added to a neuron's synaptic response current, membrane voltage, and refractory delay for solving probabilistic inference problems. Loihi can solve optimization problems such as LASSO, being over three orders of magnitude better in terms of energy-delay-product as compared to a CPU-based solver. Further, the Loihi processor is fabricated in Intel's 14 nm FinFET process.

## Discussion

The various state-of-the-art neural emulators described in this paper illustrate the evolution and advancement of neuromorphic systems and the NE field. The promising specifications and applications of these systems advance technology in a manner that further closes the gap between the computational capabilities and efficiency of the human brain and engineered systems. Moreover, it should be clearly noted that the aim of this paper is to merely elucidate each neural emulator rather than deem one inferior to another. However, depending on one's objectives and specifications of their system, one neural emulator may be more suitable than another. To briefly summarize the key points of each system, the IFAT is a mixed-signal CMOS neural array that was designed to achieve low power consumption and high neuron density (more neurons per *mm*^2^). The M–N neuron design allows for producing many different spiking behaviors, including those with an adaptive threshold. The spiking behavior of each neuron is a function of its synapses that are dynamically controlled by only a few parameters. This surpasses arrays that are limited by only an integrate-and-fire neuron model. Furthermore, this system consists of a shared synapse, threshold dynamics, and soma. Given this external circuitry (to the array, but still on-chip), each neuron consumes minimal area. Such a design permits low power, high density, and low mismatch compared to other neural array designs. HiAER-IFAT is designed by construction to run real-time at biological time scales, with average spike rates per neuron in the 10 Hz range. The HiAER hierarchical memory organization distributes the computation and communication across large numbers of cores running in parallel, where each core serves four quadrants of 16 k neurons each. The IFAT models two-compartment neurons, each with two types of conductance-based synapses, and with quadrant-shared configurable parameters for reversal potentials, leak and coupling conductances, firing thresholds, reset potentials, and synaptic time constants. Peak synaptic conductances for individual synaptic connections are stored in the HiAER memory along with the routing information for hierarchical connectivity. DeepSouth is a cortex emulator implemented on an FPGA board, and is capable of simulating up to 2.6 billion LIF neurons in real time; further, running five times slower, it can scale up to 12.8 billion LIF neuron simulations. In addition, it comes with the PyNN programming interface that will enable the rapid modeling of different topologies and configurations using the cortex emulator. The main advantages of the DeepSouth is that it uses commercial off-the-shelf FPGAs, while most of the other platforms use specialized hardware that are not easily accessible to other researchers. The DeepSouthcan be linearly scaled up using multiple FPGAs without performance loss on the back of its modular architecture and hierarchical communication scheme. The BrainScaleS neuromorphic system is wafer-scale integration of high-speed and continuous time analog neurons and synapses. The wafer module integrates an uncut silicon wafer with 384 neuromorphic chips. It provides programmable analog parameters to calibrate neurons according to models from computational neuroscience. BrainScaleS is faster and it allows to model processes like learning and development in seconds instead of hours. Dynap-SEL is a novel mixed-signal multi-core neuromorphic processor that combines the advantages of analog computation and digital asynchronous communication and routing. It takes the benefits of memory-optimized routing, which makes the system highly scalable (Moradi et al., 2015). Resources from different chips can be easily combined and merged. The 2DIFWTAchip is an implementation of cooperative-competitive networks and consists of a group of interacting neurons that compete in response to an input stimulus. The neurons with the highest response suppress all other neurons to win the competition. The cooperative and competitive networks give it power to solve complex non-linear operations. The PARCA is a parallel architecture with resistive crosspoint array. Integration of the resistive synaptic devices into crossbar array architecture can efficiently implement the weighted sum or the matrix-vector multiplication (read mode) as well as the update of synapse weight (write mode) in a parallel manner. Transistor-channel-based programmable and configurable neural system is a neuron IC built from transistor-channel-modeled components. The IC uses a mesh-type structure of synapses, an array of configurable elements to compile the desired soma dynamics, and AER blocks that output action potentials into the synapse fabric, as well as AER blocks that input action potentials from soma blocks. Furthermore, this neuromorphic IC includes dendritic computation as well as FPAA re-configurability. The computational efficiency of dendrite computation has been demonstrated to be 1000s of times more efficient than that of most analog signal processing algorithms. Each of these neuroemulators have advantageous properties that can be utilized depending on one's objective and constraints. Although many of these systems model the dynamics of biological neurons, Neurogrid and the transistor-channel-based systems more closely resemble biological neurons in terms of their physical properties. This sub-threshold, analog representation of neurons is closely related to Carver Mead's original vision. However, many of the systems presented are still capable of emulating several prominent neuron spiking behaviors aside from integrate-and-fire (e.g., IFAT, BrainScaleS, Transistor-channel, TrueNorth, and SpiNNaker). DeepSouth, TrueNorth, and SpiNNaker take a digital approach with their neuron design. Although this may be a more robust approach, such a digital design may consume more area and power. TrueNorth has lowpower consumption even while using digital technology, but this is due to the state-of-the-art 28 nm feature-size technology that it uses. If one were constrained by power consumption, the IFAT, PARCA, or transistor-channel-based systems would be ideal, as these designs were specifically optimized for low-power consumption. However, such low-power design approaches may sacrifice speed and/or accuracy. BrainScaleS and HiAER-IFAT are ideal for high-speed applications as these systems were optimized for highspeed even when simulating large networks. BrainScaleS operates at 10,000 × biological real-time speed. HiAER-IFAT uses a novel hierarchical AER design that allows for efficient transmitting and receiving of events over long distances. If one is seeking a system that consists of a well-developed, user-friendly software interface for programming and visualization of the network, the Neurogrid, SpiNNaker, DeepSouth, or TrueNorth might be the appropriate choice. Finally, one may select a system based on a very specific application. For example, for a WTA application (i.e., visual saliency application), one may take advantage of the 2DIFWTA system. If one is interested in on-chip learning, the Dynap-SEL may be ideal. The Dynap-SEL consists of circuitry designed for on-chip learning and further has proven useful as a general-purpose neuron array. This chip was designed in state-of-the-art 28 nm FDSOI technology that further makes it more advantageous with respect to neuron density and lowpower consumption.

These emulators are further compared and contrasted in the following paragraphs with respect to various aspects such as analog/digital implementations, real-time performance, and abstraction level of mimicking the brain.

The neuromorphic emulators have been implemented either with analog or digital design techniques. Each approach has its own advantages. Analog implementation is more energy-efficient and consumes less area. It exhibits high susceptibility to process mismatch in the subthreshold region. In contrast, digital implementation is immune to process variations, exhibits inherent noise rejection capacity, and exploits the advantage of technology scaling. TrueNorth, SpiNNaker, and Loihi are fully-digital designs, while Neurogrid, Dynap-SEL, BrainScaleS, HiAER-IFAT, MN-IFAT, and Transistor-channels are mostly mixed-signal VLSI implementations that include analog for neural spike computation and digital for routing the spike transmission, taking advantage of both the technology design domains. Further, DeepSouth is an FPGA-based implementation that exploits high-speed digital memories and high-speed digital communication interconnects for spike transmission and offers flexibility for rapid prototyping. The common criticism of analog-based subthreshold operation is the high degree of noise, which may limit performance and leave little scope for flexibility as the device parameters are fixed during the fabrication. Analog, however, allows a higher integration density than digital chips, although digital chips take advantage of technology scaling and squeeze more transistors to scale the performance. Most of the digital neuromorphic implementations have their software models available, which helps to tune the learning parameters during training.

TrueNorth, Neurogrid, HiAER-IFAT, and DeepSouth come with precomputed weights and currently do not offer any on-chip synaptic plasticity. Further, SpiNNaker and Loihi can be programmed for the variability of plasticity rules. BrainScaleS offers Hebbian learning, STDP, and cortical dynamics for online learning. Dynap-SEL offers Short-term plasticity (STP) plasticity rules for each synapse and also comes with a large memory chip, where individual synapses act as both memory elements and computational blocks (Moradi et al., 2018). Further, the Dynap-SEL is said to be the only existing neuromorphic system that features on-chip on-line learning with analog neurons and digital synapses in a 28 nm technology node. BrainScaleS comes with an acceleration factor of 10,000 with respect to biological real-time operations, which can be used to accelerate experimental studies. PARCA implements SGD for RRAM-based dictionary update in sparse coding tasks (Seo et al., 2015), but general spike-based learning rules can also be implemented with the PARCA system. Transistor-channel system deploys both long-term storage and PSP generation, but also has allowed a family of LTP-, LTD-, and STDP-type learning approaches through the same device (Gordon et al., 2004).

Each emulator employs various routing strategies. Neurogrid employs tree topologies and linear grids in which AERs are broadcasted across the chip, which improves the overall communication channel bandwidth among peer-to-peer connectivity but sacrifices the flexibility to implement synaptic plasticity (Park et al., 2017). Wafer-scale integration uses a whole production wafer instead of dicing it into individual reticles through metal post-processing. It realizes connectivity by 2-D grid-based routing. TrueNorth also uses 2-D mesh architecture to connect neurons. On the other hand, SpiNNaker implements connectivity via a spanning architecture. HiAER-IFAT implements multi-tree-based extension on AER synaptic routing for dynamic reconfigurability and long-term synaptic connectivity. DeepSouth stores neurons in the minicolumn and the hypercolumns in a hierarchical fashion instead of individual peer-to-peer connections. It has been argued that the hierarchical communication scheme scales with the complexity as peer-to-peer communication significantly increases memory use (Wang et al., 2018). DeepSouth differs from HiAER-IFAT in two aspects; first, HiAER-IFAT routes each individual spike generated by neurons, while DeepSouth routes the spikes generated in one minicolumn; second, HiAER supports point-to-point connections and uses external memory to store it in LUTs, each having its own programmable parameters. A mesh architecture (or crossbar, as originally described in Hasler et al., 1994) enables the highest synapse array density, interconnecting configurable, channel-neuron model components in transistor-channel implementation.

The diversity of approaches in modeling the brain comes from different research goals. The cortex has been simulated at varying levels of scale and abstraction. BrainScaleS and SpiNNaker emulate ion-channel dynamics, while IBM's TrueNorth uses higher abstraction model of a neuron. DeepSouth exploits the modular structure of the cortex, emulating the neocortex with billions of neurons with a simple LIF neuron model. MN-IFAT models M–N neurons, which are a particularly robust design and produce different a spiking behavior required for the application (Indiveri et al., 2011). Dynap-SEL emulates biologically plausible adaptive leaky I&F neuron behaviors and synaptic dynamics using spike-based computing, using both the timing and the mean rate of the spikes. The 2DIFWTA models the WTA architecture of the neocortex. These operations are believed to be widespread in the nervous system and the WTA architecture has been proposed as a computational primitive of the canonical microcircuit of the neocortex (Douglas et al., 1989). The neuron and read circuit design in PARCA (Seo et al., 2015) mimics the integrate-and-fire functionality of the neurons in our brain, where the number of pulses (spikes) that the neuron generates is proportional to the analog weighted-sum current from the RRAM array. Transistor-channel IC implements biological model circuits for dendrites, learning synapses, soma (channel model).

Each of these systems presents a unique capability that may contribute to the growth of the NE field. These contributions collaboratively move us closer to designing the most energy-efficient, highly dense neural emulator that closely mimics the biological counterpart it is emulating.

A comparison of all these neural processors is depicted in Table 5.

**Table 5:** Comparison of event-based neural processors.

**Chip name**	**Technology**	**Process (nm)**	**Neurons type**	**No. neurons**	**No. synapse**	**Area per neuron**	**Energy per event**	**Synaptic plasticity**
MNIFAT	Mixed Signal	500	LIF/ M–N	6,120	–	1,495 μ*m*^2^	360 pJ	Programmable
DeepSouth	Digital	28	LIF	200 K	–		–	No Plasticity
Dynap-SEL	Mixed Signal	28	I&F	1,088	78,080	20 μ*m*^2^	2.8 pJ	STDP
BrainScaleS	Mixed Signal	180	AdEx IF	512	100 K	1,500 μ*m*^2^	100 pJ	Hebbian learning, STDP
2DIFWTA	Analog	350	I&F	2,048	28,672		–	No Plasticity
HiAER-IFAT board with 4 chips	Analog	90	I&F	256 K	256 M	140 μ*m*^2^	22 pJ	No Plasticity
Transistor-Channel	Analog	350	Floating Gate MOSFET	100	30,000	4 *cm*^2^	10 pJ	STDP
Neurogrid	Mixed signal	180	Adaptive Quad IF	65 K	100 M	1,800 μ*m*^2^	31.2 pJ	No Plasticity
TrueNorth	Digital	28	Adaptive Exp IF	1 M	256 M	3,325 μ*m*^2^	45 pJ	STDP
SpiNNaker	Digital	130	Programmable	16 K	16 M	–	43 nJ	STDP
Loihi	Digital	14	Adaptive LIF	130 K	130 M	0.4 *mm*^2^	23.6 pJ	Epoch-based, STDP

## Author Contributions

All authors listed have made a substantial, direct and intellectual contribution to the work, and approved it for publication.

### Conflict of Interest Statement

The authors declare that the research was conducted in the absence of any commercial or financial relationships that could be construed as a potential conflict of interest.

## Footnotes

^1^The post-processing process has proven to be reliable down to a pitch of 6 μm. Since this density was not necessary, the pitch was relaxed a bit.

^2^The CS was developed by Technical University Dresden.

^3^The connectors used are from Fujipoly, Datasheet Nr. FPDS 01-34 V6.
