# Corrigendum: Large-Scale Neuromorphic Spiking Array Processors: A Quest to Mimic the Brain

**DOI:** 10.3389/fnins.2018.00991

**Published:** 2019-01-07

**Authors:** Chetan Singh Thakur, Jamal Lottier Molin, Gert Cauwenberghs, Giacomo Indiveri, Kundan Kumar, Ning Qiao, Johannes Schemmel, Runchun Wang, Elisabetta Chicca, Jennifer Olson Hasler, Jae-sun Seo, Shimeng Yu, Yu Cao, André van Schaik, Ralph Etienne-Cummings

**Affiliations:** ^1^Department of Electronic Systems Engineering, Indian Institute of Science, Bangalore, India; ^2^Department of Electrical and Computer Engineering, Johns Hopkins University, Baltimore, MD, United States; ^3^Department of Bioengineering and Institute for Neural Computation, University of California, San Diego, La Jolla, CA, United States; ^4^Institute of Neuroinformatics, University of Zurich and ETH Zurich, Zurich, Switzerland; ^5^Kirchhoff Institute for Physics, University of Heidelberg, Heidelberg, Germany; ^6^The MARCS Institute, Western Sydney University, Kingswood, NSW, Australia; ^7^Cognitive Interaction Technology – Center of Excellence, Bielefeld University, Bielefeld, Germany; ^8^School of Electrical and Computer Engineering, Georgia Institute of Technology, Atlanta, GA, United States; ^9^School of Electrical, Computer and Engineering, Arizona State University, Tempe, AZ, United States

**Keywords:** neuromorphic engineering, large-scale systems, brain-inspired computing, analog sub-threshold, spiking neural emulator

In the original article, there were mistakes in Table 5, Comparison of event-based neural processors, as published. The area per neuron for the transistor channel was incorrectly provided as “4 cm^2^” and should be “–” (empty). The synaptic plasticity for true North was incorrectly provided as “STDP” and should be “No Plasticity.” The area per neuron for Loihi was incorrectly provided as “0.4 mm^2^” and should be “0.4 mm^2*^.” The corrected Table 5, Comparison of event-based neural processors, appears below.

**Table 5:** Comparison of event-based neural processors.

**Chip name**	**Technology**	**Process (nm)**	**Neurons type**	**#Neurons**	**#Synapse**	**Area per neuron**	**#Energy per event**	**Synaptic plasticity**
MNIFAT	Mixed Signal	500	LIF/M-N	6,120	–	1,495 μm^2^	360 pJ	Programmable
DeepSouth	Digital	28	LIF	200K	–		–	No Plasticity
Dynap-SEL	Mixed Signal	28	I&F	1,088	78,080	20 μm^2^	2.8pJ	STDP
BrainScaleS	Mixed Signal	180	AdEx IF	512	100K	1,500 μm^2^	100pJ	Hebbian learning, STDP
2DIFWTA	Analog	350	I&F	2,048	28,672		–	No Plasticity
HiAER-IFAT board with 4 chips	Analog	90	I&F	256K	256M	140 μm^2^	22 pJ	No Plasticity
Transistor-Channel	Analog	350	Floating Gate MOSFET	100	30,000	–	10 pJ	STDP
Neurogrid	Mixed signal	180	Adaptive Quad IF	65K	100M	1,800 μm^2^	31.2pJ	No Plasticity
TrueNorth	Digital	28	Adaptive Exp IF	1M	256M	3,325 μm^2^	45pJ	**No Plasticity**
SpiNNaker	Digital	130	Programmable	16K	16M	–	43nJ	STDP
Loihi	Digital	14	Adaptive LIF	130K	130M	0.4 *mm*^2^^*^	23.6 pJ	Epoch-based, STDP

* *Neurosynaptic core area with each core implements 1,024 neural units*.

The authors apologize for these errors and state that the do not change the scientific conclusions of the article in any way. The original article has been updated.

